# Ecoclimate drivers shape virome diversity in a globally invasive tick species

**DOI:** 10.1093/ismejo/wrae087

**Published:** 2024-05-15

**Authors:** Xue-Bing Ni, Yao Pei, Yong-Tao Ye, Marcus Ho-Hin Shum, Xiao-Ming Cui, Yu-Qian Wu, Mac P Pierce, Lin Zhao, Gong-Pei Wang, Jia-Te Wei, Jing-Li Fan, Qian Wang, David K Smith, Yi Sun, Li-Feng Du, Jie Zhang, Jia-Fu Jiang, Pei-Jun He, Xin Chen, Hua Wei, Ning-Qi Zhao, Wu-Chun Cao, Tommy Tsan-Yuk Lam, Na Jia, Tick Genome and Microbiome Consortium

**Affiliations:** State Key Laboratory of Pathogen and Biosecurity, Beijing Institute of Microbiology and Epidemiology, Beijing 100071, People’s Republic of China; State Key Laboratory of Emerging Infectious Diseases and Centre of Influenza Research, School of Public Health, The University of Hong Kong, Hong Kong SAR, People’s Republic of China; Laboratory of Data Discovery for Health Limited, 19W Hong Kong Science & Technology Parks, Hong Kong SAR, People’s Republic of China; State Key Laboratory of Emerging Infectious Diseases and Centre of Influenza Research, School of Public Health, The University of Hong Kong, Hong Kong SAR, People’s Republic of China; Laboratory of Data Discovery for Health Limited, 19W Hong Kong Science & Technology Parks, Hong Kong SAR, People’s Republic of China; State Key Laboratory of Emerging Infectious Diseases and Centre of Influenza Research, School of Public Health, The University of Hong Kong, Hong Kong SAR, People’s Republic of China; Laboratory of Data Discovery for Health Limited, 19W Hong Kong Science & Technology Parks, Hong Kong SAR, People’s Republic of China; State Key Laboratory of Emerging Infectious Diseases and Centre of Influenza Research, School of Public Health, The University of Hong Kong, Hong Kong SAR, People’s Republic of China; Laboratory of Data Discovery for Health Limited, 19W Hong Kong Science & Technology Parks, Hong Kong SAR, People’s Republic of China; State Key Laboratory of Pathogen and Biosecurity, Beijing Institute of Microbiology and Epidemiology, Beijing 100071, People’s Republic of China; Research Unit of Discovery and Tracing of Natural Focus Diseases, Chinese Academy of Medical Sciences, Beijing 100071, People’s Republic of China; State Key Laboratory of Emerging Infectious Diseases and Centre of Influenza Research, School of Public Health, The University of Hong Kong, Hong Kong SAR, People’s Republic of China; Laboratory of Data Discovery for Health Limited, 19W Hong Kong Science & Technology Parks, Hong Kong SAR, People’s Republic of China; State Key Laboratory of Emerging Infectious Diseases and Centre of Influenza Research, School of Public Health, The University of Hong Kong, Hong Kong SAR, People’s Republic of China; Laboratory of Data Discovery for Health Limited, 19W Hong Kong Science & Technology Parks, Hong Kong SAR, People’s Republic of China; State Key Laboratory of Pathogen and Biosecurity, Beijing Institute of Microbiology and Epidemiology, Beijing 100071, People’s Republic of China; Institute of EcoHealth, School of Public Health, Shandong University, Jinan 250012, Shandong, People’s Republic of China; Laboratory of Data Discovery for Health Limited, 19W Hong Kong Science & Technology Parks, Hong Kong SAR, People’s Republic of China; Centre for Immunology & Infection Limited, 17W Hong Kong Science & Technology Parks, Hong Kong SAR, People’s Republic of China; Beijing Friendship Hospital, Capital Medical University, Beijing 100083, People’s Republic of China; Center for Sustainable Development and Energy Policy Research (SDEP), School of Energy and Mining Engineering, China University of Mining and Technology, Beijing 100083, People’s Republic of China; State Key Laboratory of Pathogen and Biosecurity, Beijing Institute of Microbiology and Epidemiology, Beijing 100071, People’s Republic of China; State Key Laboratory of Emerging Infectious Diseases and Centre of Influenza Research, School of Public Health, The University of Hong Kong, Hong Kong SAR, People’s Republic of China; Laboratory of Data Discovery for Health Limited, 19W Hong Kong Science & Technology Parks, Hong Kong SAR, People’s Republic of China; State Key Laboratory of Pathogen and Biosecurity, Beijing Institute of Microbiology and Epidemiology, Beijing 100071, People’s Republic of China; State Key Laboratory of Pathogen and Biosecurity, Beijing Institute of Microbiology and Epidemiology, Beijing 100071, People’s Republic of China; State Key Laboratory of Pathogen and Biosecurity, Beijing Institute of Microbiology and Epidemiology, Beijing 100071, People’s Republic of China; State Key Laboratory of Pathogen and Biosecurity, Beijing Institute of Microbiology and Epidemiology, Beijing 100071, People’s Republic of China; Research Unit of Discovery and Tracing of Natural Focus Diseases, Chinese Academy of Medical Sciences, Beijing 100071, People’s Republic of China; State Key Laboratory of Pathogen and Biosecurity, Beijing Institute of Microbiology and Epidemiology, Beijing 100071, People’s Republic of China; School of Public Health and Health Management, Gannan Medical University, Ganzhou 341000, People’s Republic of China; School of Public Health and Health Management, Gannan Medical University, Ganzhou 341000, People’s Republic of China; Institute of EcoHealth, School of Public Health, Shandong University, Jinan 250012, Shandong, People’s Republic of China; Laboratory of Data Discovery for Health Limited, 19W Hong Kong Science & Technology Parks, Hong Kong SAR, People’s Republic of China; State Key Laboratory of Pathogen and Biosecurity, Beijing Institute of Microbiology and Epidemiology, Beijing 100071, People’s Republic of China; Research Unit of Discovery and Tracing of Natural Focus Diseases, Chinese Academy of Medical Sciences, Beijing 100071, People’s Republic of China; Institute of EcoHealth, School of Public Health, Shandong University, Jinan 250012, Shandong, People’s Republic of China; The representative of Tick Genome and Microbiome Consortium (TIGMIC); State Key Laboratory of Emerging Infectious Diseases and Centre of Influenza Research, School of Public Health, The University of Hong Kong, Hong Kong SAR, People’s Republic of China; Laboratory of Data Discovery for Health Limited, 19W Hong Kong Science & Technology Parks, Hong Kong SAR, People’s Republic of China; Centre for Immunology & Infection Limited, 17W Hong Kong Science & Technology Parks, Hong Kong SAR, People’s Republic of China; Guangdong-Hongkong Joint Laboratory of Emerging Infectious Diseases, Joint Institute of Virology (Shantou University/The University of Hong Kong), Shantou 515063, Guangdong, People’s Republic of China; EKIH (Gewuzhikang) Pathogen Research Institute, Futian District, Shenzhen 518045, Guangdong, People’s Republic of China; State Key Laboratory of Pathogen and Biosecurity, Beijing Institute of Microbiology and Epidemiology, Beijing 100071, People’s Republic of China; Research Unit of Discovery and Tracing of Natural Focus Diseases, Chinese Academy of Medical Sciences, Beijing 100071, People’s Republic of China

**Keywords:** ecological modeling, deep learning network, tick virome, tick-borne viruses, climate changes, meta-transcriptomics

## Abstract

Spillovers of viruses from animals to humans occur more frequently under warmer conditions, particularly arboviruses. The invasive tick species *Haemaphysalis longicornis*, the Asian longhorned tick, poses a significant public health threat due to its global expansion and its potential to carry a wide range of pathogens. We analyzed meta-transcriptomic data from 3595 adult *H. longicornis* ticks collected between 2016 and 2019 in 22 provinces across China encompassing diverse ecological conditions. Generalized additive modeling revealed that climate factors exerted a stronger influence on the virome of *H. longicornis* than other ecological factors, such as ecotypes, distance to coastline, animal host, tick gender, and antiviral immunity. To understand how climate changes drive the tick virome, we performed a mechanistic investigation using causality inference with emphasis on the significance of this process for public health. Our findings demonstrated that higher temperatures and lower relative humidity/precipitation contribute to variations in animal host diversity, leading to increased diversity of the tick virome, particularly the evenness of vertebrate-associated viruses. These findings may explain the evolution of tick-borne viruses into generalists across multiple hosts, thereby increasing the probability of spillover events involving tick-borne pathogens. Deep learning projections have indicated that the diversity of the *H. longicornis* virome is expected to increase in 81.9% of regions under the SSP8.5 scenario from 2019 to 2030. Extension of surveillance should be implemented to avert the spread of tick-borne diseases.

## Introduction

Recent climate changes leading to warmer temperatures are thought to increase the possibility of more cross-species transmission events by viruses, especially arboviruses, which are considered capable of infecting wider ranges of mammalian hosts than other viruses [1, 2]. Manifestations of climate sensitivity span pathogens and vectors, with changes in climate having varied effects on arthropod vector populations and their viruses. These effects include biological impacts such as enhanced development rate, increased population size, and expanded geographic range, as well as behavioral impacts including enhanced biting rate, broadened host preference, and temperature induced host-seeking activity [3–8]. Climate change can also affect the dynamics of arbovirus infections. For example, warmer temperatures may facilitate amplified virus proliferation rates as well as elevated rates of infections with tick-borne viral illnesses, including Crimean-Congo hemorrhagic fever, tick-borne encephalitis, and severe fever with thrombocytopenia syndrome (SFTS) [9–11]. Considering that most climate-sensitive diseases, 41% of which are tick borne, are transmitted by arthropods [8], collectively their impacts may lead to more opportunities for cross-species transmission of arboviruses.

Current research has focused largely on the impacts of climate change in the context of single specific disease-causing viruses, with studies conducted under simulated climate parameters [9, 10]. However, actual long-term changes in climate have not shown a consistent observable pattern, a situation that leads to multifaceted nonlinear effects on viruses in terms of the variation of individual viruses, their community inside a vector, and their interactions with different viruses, all of which have impacts on their potential for emergence [12, 13]. Therefore, developing a holistic perspective of how climate influences a viral community within a vector under natural conditions will provide broader insights regarding arbovirus dynamics and the potential risks they pose to ecosystems.

The invasive species *Haemaphysalis longicornis* is a significant emerging threat to public health due to its increasing global distribution and ability to carry a high diversity of pathogens*. H. longicornis* native habitats are in eastern and central Asia, but it has successfully invaded Australia, New Zealand, the Pacific Islands, and the United States, probably because it is capable of parthenogenetic reproduction, whereby a single female tick can generate a population [14–19]. Predictive modelling suggests that the future habitable range of *H. longicornis* will span all continents except Antarctica, including regions that lie between latitudes 18°–53° north and 16°–45° south [18], with coastal areas being most suitable [18]. *H. longicornis* is also a competent vector capable of transmitting a high diversity of pathogens, including the Powassan virus, Khasan virus, tick-borne encephalitis virus, and SFTS virus (SFTSV), the incidence and range of which have increased to the detriment of human health and animal welfare [20–22]. Beyond the public health burden imposed by *H. longicornis* due to its vector competence and broad climate envelope, *H. longicornis* may be considered a suitable model of a virus vector for studying climate change impacts on virome–vector dynamics.

In this study, the associations between ecological factors, climate factors, and virome diversity within *H. longicornis* ticks from China were examined, with a particular emphasis on viruses capable of cross-species transmission among vertebrate hosts. Meta-transcriptomic sequencing was utilized to characterize the viral community and generate estimates of virome diversity. Generalized additive modelling (GAM) results were subsequently analyzed to identify ecoclimate determinants that might be involved in shaping the diversity of vertebrate-associated viruses and assess their associations. Causality analysis was incorporated to gain a better understanding of their causal relationships. Finally, a deep learning (DL) model was employed to forecast changes in vertebrate-associated virome diversity globally through the coming decade under different paradigms of atmospheric carbon dioxide levels.

## Materials and methods

### Sample preparation and sequencing

Adult *H. longicornis* ticks were collected by flag-dragging or from animal hosts from 22 provinces of China, covering steppe, farmland, desert, shrubland, and forest. The related geographic and ecological characteristics, i.e., latitude, longitude, tick gender, and tick host animals, were recorded.

Ticks were sterilized by washing twice in 70% ethanol for 30 seconds and homogenized in RLT solution under liquid nitrogen. RNA extraction was performed using the AllPrep DNA/RNA Mini Kit (Qiagen, United States) and sent for transcriptome sequencing (RNA-seq). After rRNA removal and sequencing library preparation, paired-end (2×150 bp) sequencing was conducted on a HiSeq 4000 platform (Illumina) at Novogene Tech (Beijing, China).

### Analyses of vertebrate-associated virome diversity

Clean reads were generated after adapter removal and quality check using Trimmomatic v0.39 [23]. Virus genomes were recovered by *de novo* assembly using Trinity v2.8.5 [24]. Assembled contigs were filtered based on the results of BLASTN on the bacteria and host nonredundant nucleotide databases with a cutoff of 85% identity [25]. Taxonomic classifications were performed by comparing the contigs to the RNA virus nucleotide and protein databases from NCBI using local implementations of BLAST programs [25]. The pairwise identities of downloaded reference amino acid sequences were calculated against all references of the same arbovirus family (*Phenuiviridae*, *Rhabdoviridae*, *Flaviviridae*, *Chuviridae*, *Nodaviridae*, *Reoviridae*, *Nairoviridae*, *Orthomyxoviridae*, *Peribunyaviridae*) using BLASTP [25]. The Q1–1.5 IQRs of pairwise identities were set as the cutoff for each viral family. Those virus contigs showing more than their corresponding identity cutoff to any virus species of the arboviruses families listed above were extracted to determine quantification of viral abundance. The 90% amino acid similatirity was set to the cutoff of determining viral contigs to be virus or virus like from the extracted BLASTX results mentioned above. The non-rRNA reads from each library were mapped against the identified arbovirus sequences using a Bowtie2 local alignment with very sensitive parameters [26]. Mapped sequences were grouped into operational taxonomic units (OTUs) based on 95% nucleotide identity determined by CD-HIT v4.8.1 [27]. The abudance of each OTU was summarised as the read counts of all sequences of the same OTU and normalized using TPM (transcripts per million). The Shannon index, Simpson index, ACE, Chao1, Simpson E index, and McIntosh E index were computed with the Python package “skbio” (http://scikit-bio.org/) based on the relative abundance. Statistical differences in alpha diversity among groups of different ecotypes were accessed with the Mann–Whitney U-test or Kruskal-Wallis test using SPSS version 20.0 [28]. The viral abundance and prevalence of reported human pathogenic viruses were summarized according to ecotypes.

The shapefiles of the South China Sea, East China Sea, Yellow Sea, Persian Gulf, Gulf of Thailand, Japan Sea, Andaman Sea, Arabian Sea, Bay of Bengal, Laccadive Sea (under International Hydrographic Organization Sea Area place type), and Caspian Sea (under Worldlake place type) were downloaded from Marineregions.org (https://www.marineregions.org). The latitude and longitude coordinates of the downloaded coastlines were extracted from the shapefiles using the *sf*, *sp*, and *methods* packages in R 3.6.1 [29]. The distance from each sample site to the downloaded coastline was calculated according to the Haversine method (radius of earth = 6371 km) using the *geosphere* package in R.3.6.1 [30]. The shortest distance was selected as the distance to the closest coastline. Coastline grouping was performed from 100 to 600 km with a step size of 100 km. Diversities of the vertebrate-associated virome of each coastline-distance group were compared by using the Mann–Whitney U-test and Kruskal-Wallis test.

### Tick immunity pathway analysis

The assembly contigs for all RNA-sequencing datasets were annotated according to the *H. longicornis* reference genome (GWHAMMI00000000). The antiviral immunity–related genes were extracted and used as the reference dataset for aligning reads back to estimate their expression level with Bowties2 v2.4.3 [26]. The relative abundance of antiviral immunity genes was obtained by performing the fragments per kilobase of transcript per million mapped reads normalization.

### Ecoclimate predictors preparation

Climate factors were obtained from the China National Meteorological Center according to the sampling coordinates and collection date of each library. Mammal host biodiversity and domestic animal density data were downloaded from the Global Mammal Assessment database (https://globalmammal.org/) and the Gridded Livestock of the World database (https://dataverse.harvard.edu/dataverse/glw), respectively. We summed domestic animal headcount (poultry, goat, buffalo, cattle, sheep, and pig) into a single predictor (Shannon index as representative of livestock mammal biodiversity) using the Python package “skbio” (http://scikit-bio.org/). Mammal host biodiversity and livestock mammal biodiversity were assigned to each library by identifying the nearest coordinates compared with the above two global databases.

### Association between virome and occurrence of pathogenic viruses

We conducted a meta-review for occurrences of tick-borne pathogenic virus from sequence information in GenBank and published *H. longicornis* studies in PubMed. Missing coordinates were extracted for each record using the “Geocoding” API from Google Maps. We summed the species number of tick-borne pathogenic viruses (TBPVs) around the coordinate of each RNA-sequencing dataset with a radius of 50, 100, or 150 km. We divided the TBPV occurrence into three group: non-TBPV occurrence, single-TBPV occurrence, and multiple-TBPV occurrence if more than one TBPV species was reported around the RNA-sequencing dataset. Virome Shannon index values were compared among the three groups with the nonparametric Mann–Whitney U-test and Kruskal-Wallis test.

### GAM fitting for accessing climatic factors

A generalized additive model was applied to study the effects of eight meteorological factors (monthly mean temperature, maximum temperature, minimum temperature, air pressure, wind speed, relative humidity, average precipitation, and maximum precipitation from 2016 to 2019) and other ecological variables (ecotypes, geographic closeness to coastline, feeding host species, mammal biodiversity, livestock biodiversity, tick antiviral immunity, tick gender) on the diversity of the vertebrate-associated virome in *H. longicornis*. The full dataset was randomly split into a 90% training set and a 10% leave-out testing set. A variance inflation factor was used to remove factors showing colinearity (those larger than 10 were removed). The variable minimum temperature was removed due to its significant colinearity observed in both the full dataset and the training set. The overall intersectional and specific individual effects from the selected variables were fitted using the GAM to examine the diversity of the vertebrate-associated virome with respect to these factors (REML [restricted maximum likelihood] method) [31] according to their explained deviances by use of the *MGCV* package (version 1.8-12) in R 3.6.1 [32]. Categorical and binary variables were fitted as random effects of each variable level. Five-fold cross-validation was applied for model optimization on the training set. A quasipoisson GAM was used to analyze the effects of the selected variables on explanations of the change in the relative abundances of pathogenic viruses, as the relative abundances are all positive values. The leave-out testing dataset was used as an independent dataset to evaluate the final performance of the best model after it had been trained and tuned using the training data, and to estimate how well it would generalize to unseen data.

Causality effects were analyzed between the virome diversity index and ecoclimate factors by using the LiNGAM method from the pcalg package (version 2.7-9) [33]. We randomly subsampled 90% of our dataset 100 times and estimated the 95% confidence interval (CI) for the causality effect power.

### Deep learning model for global virome diversity maps

A DL model, comprising a deep neural network, was developed for predicting vertebrate-associated virome diversity in *H. longicornis* based on the six climate factors (mean temperature, maximum temperature, air pressure, wind speed, relative humidity, and average precipitation) that have been used in the GAM. The model had a sequential architecture consisting of five fully connected (dense) layers to eliminate overfitting (238 samples in this study) [34]. The best predictive model, with a prediction accuracy of 0.75, had 128, 256, 256, 256, and 1 neurons in each dense layer. The model was optimized with the Adam algorithm (learning rate = 0.001) using a mean absolute error (MAE) loss function [35]. Five-fold cross-validation was applied for model optimization on the training set used for the GAM model (90% of the data randomly split from the full dataset). The best model was selected from 50 training models using 10 000 epochs each with a minibatch size of 128. The performance of the best selected model was evaluated on the leave-out dataset.

Global climate data, including near-surface air temperature (“tas”, ), daily maximum near-surface air temperature (“tasmax”, ), sea level pressure (“psl”, Pa), precipitation (“pr”, kg m-2 s-1), near-surface relative humidity (“hurs”, %), and near-surface wind speed (“sfcWind”, (m s-1), under three shared socioeconomic pathway (SSP) scenarios (SSP2.6, SSP4.5, and SSP8.5) of the CNRM-CM6–1-HR climate model [36] from year 2016 to year 2030 were obtained from the World Climate Research Programme Coupled Model Intercomparison Projects (CMIP6) [37, 38]. The data had a spatial resolution of 50 km and were downloaded in a monthly temporal frame. The units of precipitation were converted from kg m-2 s-1 to mm. Each climate variable was then transformed into a 4-year average value. For example, for the year 2019, the 4-year average value of each variable was calculated using the data from the years 2016, 2017, 2018, and 2019. Effect sizes among different years or SSPs were estimated by using the R package “effsize” (version 0.8.1).

## Results

### Ecological environment affecting tick virome diversity

**Figure 1:**
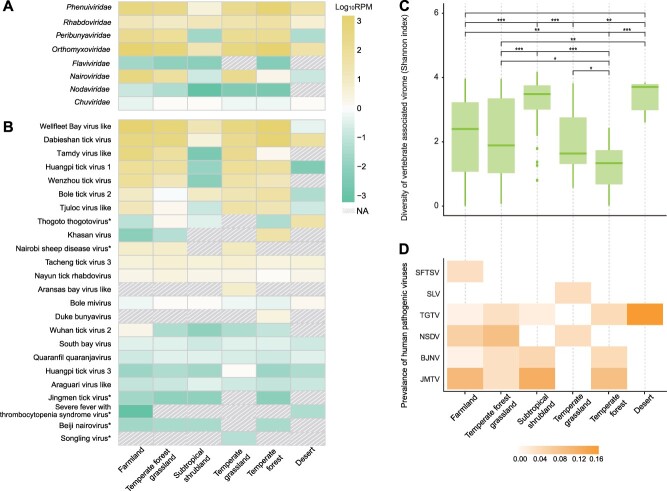
Vertebrate-associated virome of *Haemaphysalis longicornis* in different ecotypes. (A) Mean relative abundance of each vertebrate-associated viral family in different ecotypes. (B) Mean relative abundance of each vertebrate-associated viral species in different ecotypes. Human pathogenic viruses are indicated by asterisk. The mean relative abundance has been normalized by reads per million (viral reads number × 10^6^/total reads number) and taken to Log10 for better visualization. Yellow color represents higher mean viral abundance, whereas green represents lower abundance. (C) Diversity of vertebrate-associated viromes (Shannon index) in different ecotypes. ^*^^*^^*^P < .001; ^*^^*^P < .01; ^*^P < .05; ns indicates *P* > .05. (D) Prevalence of each human pathogenic virus in different ecotypes. Severe fever with thrombocytopenia syndrome virus (SFTSV), Songling virus (SLV), Tacheng tick virus (TGTV), Jingmen tick virus (JMTV), Beiji nairovirus (BJNV), and Nairobi sheep disease virus (NSDV).

A total of 3595 *H. longicornis* ticks were collected from 20 provinces in mainland China during 2016–2019. Six ecotypes (farmland, subtropical shrubland, temperate forest grassland, temperate grassland, temperate forest, and desert) were sampled. A meta-virome sequencing approach was applied to 238 RNA libraries to recover viral genomes and quantify viral abundance (Table S1). *Orthomyxoviridae* was the most abundant virus family in five ecotypes, with *Phenuiviridae* most abundant in deserts. At the species level, the Wellfleet Bay virus was like the most abundant outside of deserts, where the Thogoto thogotovirus was most abundant (Fig. 1A and B). *H. longicornis* ticks collected from desert and subtropical shrubland were found to carry the greatest diversity (Shannon index) of vertebrate-associated viruses (Wilcoxon signed-rank test, *P* < .05, Fig. 1C). The prevalence of viruses pathogenic to humans was high in temperate forest grassland (20.4%), subtropical shrubland (19.6%), and farmland (19.5%) (Fig. 1D). Ticks collected from farmland had five species of pathogenic viruses, including Jingmen tick virus, Beji nariovirus, Nairobi sheep disease virus, Thogoto thogotovirus, and SFTSV, which potentially pose a higher risk of infection to animals and humans in that ecosystem (Fig. 1D).

**Figure 2:**
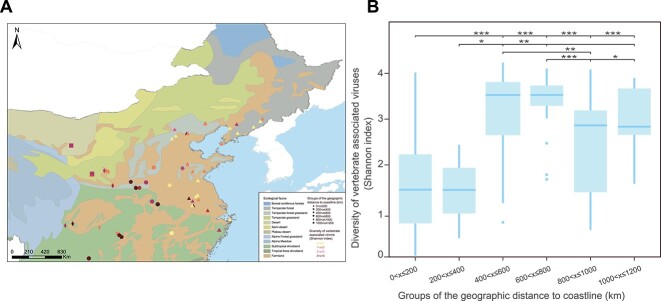
(A) Geographic map of sampling sites and the diversity of the vertebrate-associated virome (Shannon index) at that site.-(B) Vertebrate-associated virome diversity (Shannon index) grouped by geographic closeness to the coastline. ^*^^*^^*^P < .001; ^*^^*^P < .01; ^*^P < .05; ns *P* > .05.

As coastline regions have been considered suitable habitats for *H. longicornis* [18, 39], the 238 libraries were classified into groups according to their distance from the nearest coastline. Several incremental grouping definition strategies were tested, using distance ranges from 100 to 600 km, with increments of 100 km. The grouping strategy based on a step-size of 200 km yielded the most significant difference of vertebrate-associated viruses among groups based on a Kruskal–Wallis rank-sum test (Tables S2 and S3) and also had the highest explained variance for virus diversity, suggestive of the highest effect from a GAM model (Table S2). Groups located within 400 km from the nearest coastline (0–200 and 200–400 km) had significantly lower virome diversity compared to more distant groups (Wilcoxon signed-rank test, adjusted *P* < .05, Fig. 2A and B), irrespective of the distribution of low-diversity habitats (Fig. S1).

### GAM modelling and causality analysis for the association between ecoclimate factors and tick virome

Ecotypes and geographic closeness to the nearest coastline, both of which would be expected to have different climatic conditions, had significantly different vertebrate-associated virome diversity. Therefore, a generalized additive model was applied to further infer the effects of ecoclimate factors on vertebrate-associated virome diversity in *H. longicornis*. Six meteorological factors (mean temperature, maximum temperature, air pressure, wind speed, relative humidity, and precipitation [Fig. S2]), ecological variables (ecotypes, geographic closeness to coastline), biological variables (gender and antiviral immunity), and host-related variables (feeding host species, mammal biodiversity, livestock biodiversity) after removal of colinear variables, were included for GAM fitting.

**Figure 3:**
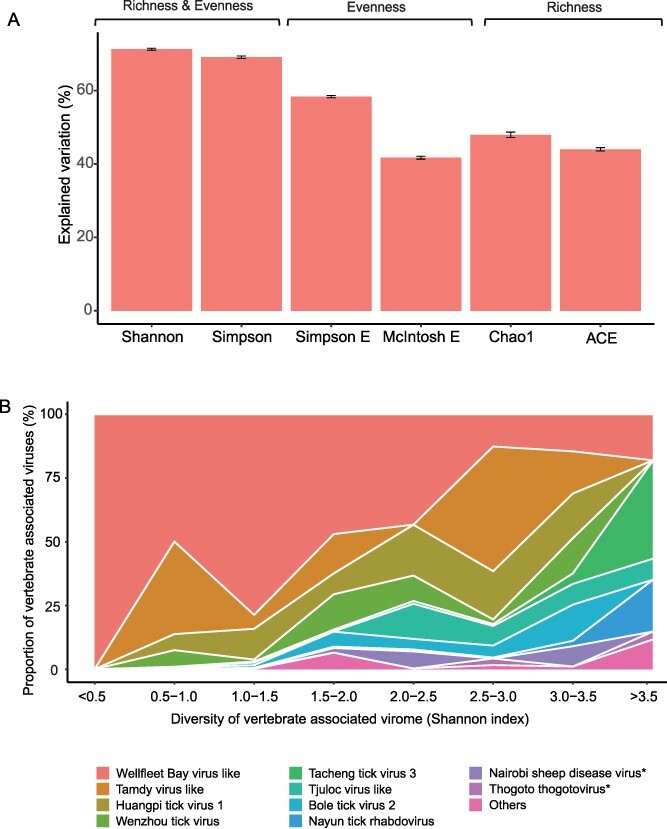
(A) Explained variation of ecoclimate factors by GAM under six index measurements of virome diversity. All ecoclimate factors were included to fit a joint model for six virome diversity index values, successively. (B) Proportions of the 10 virus species with the largest abundance in different virome diversity groupings (by Shannon index). Pathogenic virus is indicated by asterisk. ^*^^*^^*^P < .001; ^*^^*^P < .01; ^*^P < .05.

Among six diversity indices tested, the Shannon index showed the strongest effect and was chosen for use in downstream analyses (Fig. 3A). Viral evenness (Simpson E index and McIntosh E) revealed higher explained variance from GAM models (Fig. 3A) and higher correlation with higher Shannon index than richness (linear regression, *P* < .05, Fig. S3, Table S4). These results suggested that climate-induced factors may have a major impact on the evenness of virome structure. The Wellfleet Bay virus like was mostly dominant in samples of low Shannon index, whereas its viral abundance significantly decreased as Shannon index increased (Pearson coefficient: −-0.75, *P* < .05, Fig. 3B and Table S5). Besides, it showed significant negative association with other resident virus (*P* < .05, Table S4), especially pathogenic viruses including Nairobi sheep viruses and Thogoto thogotovirus, whose abundance significantly increased in samples of high Shannon index (*P* < .05, Fig. 3B and Table S5). The observed negative correlation in abundance of the Wellfleet Bay virus like and other resident viruses suggests that changes in virome structure may be associated with interactions between viruses (Fig. 3B).

**Figure 4:**
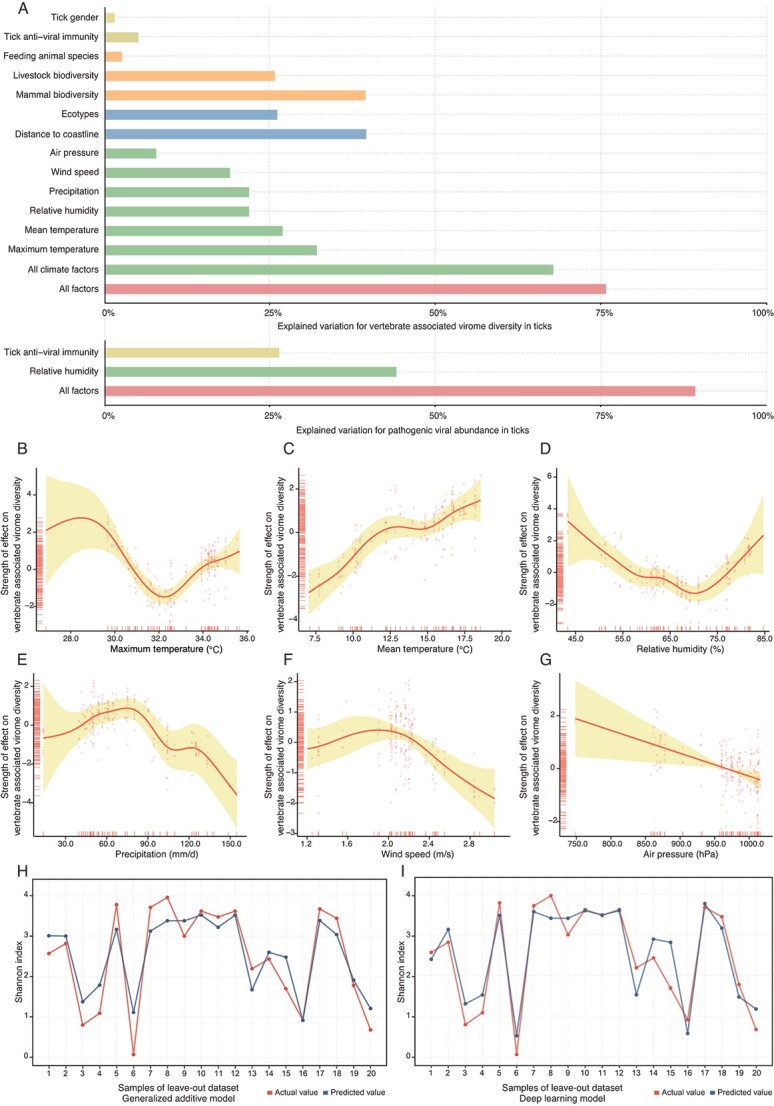
The effects of ecoclimate factors that explain the vertebrate-associated virome diversity and viral abundance of human pathogenic viruses under the GAM. (A) Explained variation of vertebrate-associated virome diversity and human pathogenic viral abundance for all ecoclimate factors. Each ecoclimate factor was individually fitted to the model for vertebrate-associated virome diversity or pathogenic viral abundance. (B-G) Partial effect plots showing the relative effect of each variable for vertebrate-associated virome diversity (Shannon index). All ecoclimate factors were included to fit the best-fit GAM model. Shaded area is the 95% confidence interval of the mean partial effect. (H) Actual Shannon index and predicted Shannon index using the GAM model. (I) Actual Shannon index and predicted Shannon index using the deep learning model.

Each categorical individual factor was fit to a GAM to access their importance by strength (percentage of deviance explained in the change in Shannon index). Geographic closeness to a coastline (deviance explained: 39.6%, Fig. 4A) and ecotypes (deviance explained: 26.1%, Fig. 4A) had a higher contribution to the explained variance of virome diversity, indicating that climate variations derived from different geographical conditions (the six ecotypes and distance from coastline) might also have an impact on the tick-borne virome. Host ranges and hematophagy have been considered to be the key contributors in shaping their vertebrate-associated virome [5]; thus animal biodiversity (deviance explained: 39.5%, Fig. 4A) and domestic animal diversity (deviance explained: 25.7%, Fig. 4A) showed important impacts from GAM modelling. The reason might be related to *H. longicornis* as a generalist that can feed on a broad range of land animals and may have significant associations with the host range [18]. Other biological factors including tick gender, antiviral immunity response, and feeding host species had a minor role in the GAM model analysis (Fig. 4A).

We furthered assessed the association between meteorological factors and virome diversity. GAM fitting of 6 meteorological factors explained 68% of the total deviance, with maximum temperature ranked as the most important predictor (deviance explained: 32.1%, Fig. 4A), followed by average temperature (deviance explained: 26.9%, Fig. 4A). The response curve for maximum temperature below 29°C or above 32°C and increasing mean temperature overall suggested a positive effect on increasing virome diversity (Fig. 4B and C), whereas virome diversity appeared to decrease for maximum temperatures between 29°C and 32°C (Fig. 4B). Relative humidity showed a minimum of diversity at 70%; precipitation, wind speed (after early maxima), and air pressure (overall) showed negative associations with vertebrate-associated virome diversity (Fig. 4D–G). The leave-out dataset demonstrated an explained variance of 0.88 with an MAE of 0.32 and showed consistent variation between actual and predicted values (Fig. 4H).

**Figure 5:**
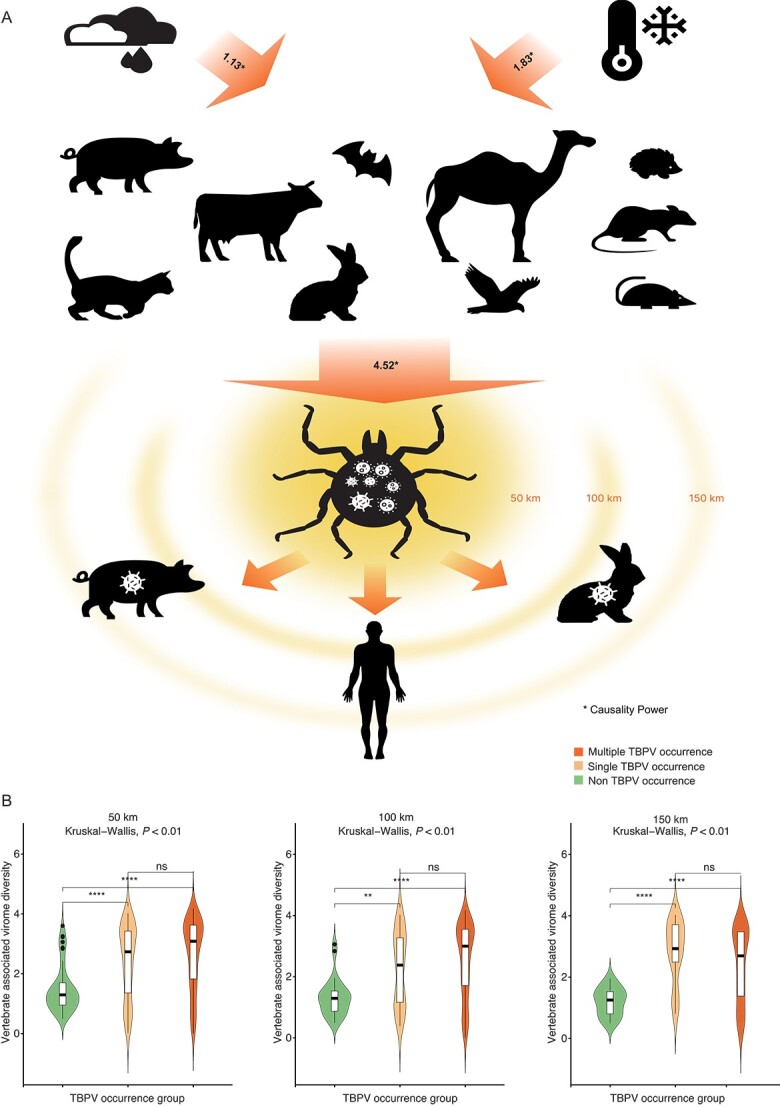
The causality mechanism of ecoclimate factors that have an impact on the vertebrate-associated virome diversity. (A) Causality effect of ecoclimatic factors on vertebrate-associated virome diversity. The number on the arrow indicates the power of the causality effect of humidity and minimum temperature on mammals, and mammals on the tick virome. Causality power for 90% subset dataset: 95% CI: [0.50, 0.59] for humidity; [1.00, 1.06] for minimum temperature; [3.06, 3.16] for mammals. (B) Comparing virome diversity in regions with varying tick-borne pathogenic virus occurrences (multiple occurrences, single occurrence, and non–tick-borne pathogenic virus) within a 50-, 100-, and 150-km radius.

Following GAM analysis, we used the LiNGAM causality test (a linear model that distinguishes between cause and effect) to identify the strength and significance of causal linkages in the environment–host–virome network. The causality analysis showed that humidity (coefficient: 1.13) and minimum temperature (coefficient: 1.60) lead to variation of animal host diversity. Animal host diversity had causality effects (coefficient: 4.52) on vertebrate-associated virome diversity (Fig. 5A). Based on the meta-review for TBPV cases and comparison of Shannon index among different TBPV occurrence groups, we found that those areas with single or multiple TBPV occurrences had higher vertebrate-associated virome diversity (Shannon index) than non–TBPV occurrence areas with a radius of 50 , 100, and 150 km (Fig. 5B). This finding revealed the mechanism that climate influences virome through direct effects on the diversity of animal hosts. Moreover, it emphasizes the public health significance of regions with higher diversity, as these areas tend to experience a higher number of tick-borne virus cases.

### GAM modelling of ecological factors with viral abundance

Six species of pathogenic viruses were detected from 43 libraries. The best model, fitted with relative humidity and antiviral immunity, explained 89.6% of the total deviance (Fig. 4A). Relative humidity showed the strongest association with the abundance of pathogenic viruses (deviance explained: 44.2%, Fig. 4A). Anti-viral immune response had a higher association on the abundance of pathogenic viruses (deviance explained: 26.4%, Fig. 4A) than vertebrate-associated virome diversity (deviance explained: 5.0%, Fig. 4A). Three highly prevalent viruses, including the Wellfleet Bay virus, Tacheng Tick Virus 3, and Nayun tick rhabdovirus, have been analyzed as depicted in Fig. 3B, as these viruses have a sufficient sample size for GAM modeling. The explained deviances for all factors were as follows: 95.2% for the Wellfleet Bay virus, 55.8% for the Tacheng Tick Virus 3, and 70% for the Nayun tick rhabdovirus (Table S6). Climate factors exhibited significantly high associations with viral abundance, accounting for 89.2% of the explained deviance for the Wellfleet Bay virus, 45.7% for the Tacheng Tick Virus 3, and 50.4% for the Nayun tick rhabdovirus. These findings highlight the diverse effects of ecoclimate factors on individual viruses. Specifically, the Wellfleet Bay virus appears to have a strong association with ecoclimate factors, particularly climate factors.

### Deep learning network for extension worldwide

Based on the indicators from the GAM described above, which showed the strong effect of six meteorological factors, DL networks were performed to predict global hotspots of tick-borne virus diversity. The DL model was trained on the 238 datasets with the best 75% accuracy and used to forecast global hotspots. The leave-out dataset demonstrated an explained variance of 0.93 with a MAE of 0.075 and consistent variation (Fig. 4I), indicating that climate factors have substantial predictive performance in the tick virome. Eight sets of publicly available RNA-sequencing data from the SRA database of NCBI from countries other than China (United States and South Korea) were also analyzed (for their vertebrate-associated virome diversity) and were used to assess the accuracy of predictions resulting from the DL model. Both the actual Shannon index and the predicted Shannon index for each dataset were lower than the corresponding overall mean Shannon index (averaged Shannon index of all locations of the corresponding sampling year), suggesting consistency in identifying the level of diversity of regions through DL–based model prediction (Table S7).

**Figure 6:**
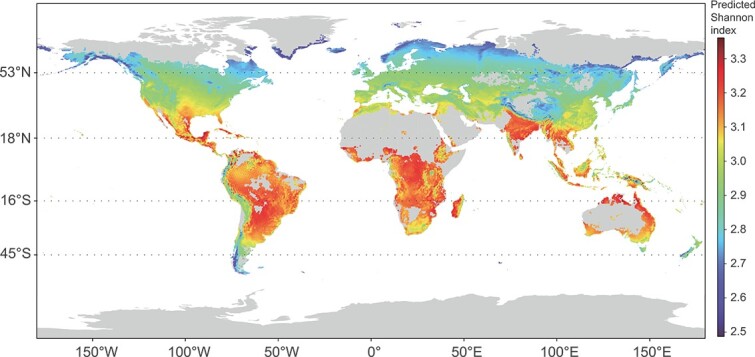
Global prediction of vertebrate-associated virome diversity under SSP4.5 in 2019.

Under three separate SSP scenarios (SSP 2.6, 4.5, and 8.5), regions predicted to have higher tick-borne virus diversity than the overall average predicted virus diversity included Southeast Asia, in particular India; Thailand; Myanmar; coastal areas of Malaysia and Indonesia; the northern coastline of Australia; multiple areas in the New World, including the western coastline of the United States, coastal areas of Mexico, and the majority of South America; and the most part of Sub-Saharan Africa, as well as Madagascar. Though much of the area predicted to have hotspots of high tick-borne virus diversity occurs in the tropics, there is significant overlap with regions between 18° and 53° north latitude and 16° and 45° south latitude, previously predicted to be suitable *H. longicornis* tick habitat [18] (Fig. 6, Fig. S4–S8, Table S8). The western coastline of the United States and the northeastern coastline of Australia, which have been considered as the most suitable habitats for *H. longicornis* [39], could potentially experience higher cross-species transmission in these regions due to its more diverse vertebrate-associated virome. Regions located between 45° and 53° in the Northern Hemisphere were predicted to have relatively lower risk, especially eastern Russia and the Qinghai-Tibet Plateau of Asia (Table S8).

Given that the objective of WHO SDG Target 3.3 is to eradicate epidemics of AIDS, tuberculosis, malaria, and neglected tropical diseases and combat hepatitis, water-borne diseases, and other communicable diseases by 2030, we strongly emphasize the significance of predicting tick-borne viral diversity by 2030 to enhance the prevention and control of tick-borne illnesses [40]. Virome diversity was predicted to increase from 2019 to 2030 by 0.19% (95% CI: −0.42%, 0.81%) and by 0.33% (95% CI: −0.37%, 1.03%) under the SSP4.5 and SSP8.5 scenarios, respectively, in the tick-reported regions including 289 previously reported locations and the 238 locations sampled in this study (Table S9) [18]. A decrease of 0.09% (95% CI: −0.95%, 0.75%) in the diversity of viruses was predicted under SSP2.6 (Table S8). The predicted virome diversity was observed to increase in 41.2% (95% CI: 33.9%, 48.5%), 71.2% (95% CI: 64.5%, 77.9%), and 81.9% (95% CI: 76.2%, 87.6%) of above tick reported regions under the SSP2.6, SSP4.5, and SSP8.5 scenarios, respectively. For the same sampling locations in 2030, the Shannon index increased in 73.9% (95% CI: 68.3%, 79.5%) and 76.1% (95% CI: 70.7%, 81.5%) of the locations under SSP4.5 and SSP8.5, respectively. Under SSP2.6, 30.4% (95% CI: 24.6%, 36.3%) of the locations showed an increase (Table S9). Shannon index slightly increased in above regions by 0.43% (95% CI: 0.044%, 0.99%), 0.35% (95% CI: 0.057%, 0.74%) and 0.33% (95% CI: 0.024%, 0.8%) under SSP2.6, SSP4.5 and SSP8.5, respectively. From the 2030–2019 diversity difference map (Fig. S9), the region with the largest increase in diversity is southwestern India, whereas the largest decrease is observed in the southern part of South Africa. The effect size (Cohen’s d) for diversity changes between 2030 and 2019 are −0.038 (95% CI: −0.052, −0.024) for SSP2.6, −0.034 (95% CI: −0.048, −0.019) for SSP4.5 and − 0.052 (95% CI: −0.066, −0.038) for SSP8.5, respectively. These values indicate relatively low differences between 2030 and 2019.

The recurring pattern of major pandemics (such as SARS-CoV in 2003, H1N1 in 2009 and COVID-19 in 2019) occurring approximately every 10 years, has also increased attention towards the ecological and genetic patterns of viruses over a decade. However, considering the near future of 2030, we have conducted projections for 2040 and 2050 to gain insights into the changing trend of tick virome in the more distant future. These projections indicate that virome diversity in ticks inhabiting tropical and subtropical regions between latitude 35°N and 45°S is expected to increase in both 2040 and 2050 (Fig. S10-S15).

## Discussion

In this study, comprehensive metagenomic sequencing was utilized to extend the understanding of ecoclimate impacts on viruses from the level of individual viruses to the whole viral community within a specific vector species, *H. longicornis*. Compared to primer-based PCR detection of viruses, metagenomic data can be more readily used for gauging changes in virome richness and composition, assessing interactions between viruses, vectors and environmental factors [41, 42]. Based on our understanding, this study provides novel evidence from a comprehensive virome-scale analysis, establishing a macroecological link with ecoclimate factors. We observed that climate factors exerted a more pronounced influence compared to other variables on the diversity of tick-borne vertebrate-associated virome. Of particular interest was the finding that elevated temperature and lower humidity were associated with increased diversity in animal hosts, potentially leading to variations in tick-borne vertebrate diversity. This phenomenon may be attributed to rapid environmental changes prompting animal hosts to expand their habitats through migration as a means of survival. Consequently, these findings raise the possibility of increased exposure of host animals to TBPV. carried by ticks. This may result in more frequent cross-species transmission of viruses between animal hosts and ticks, with estimates suggesting a 4000-fold increase compared to previous levels [1]. Our findings illuminate how climate impacts the virome through influencing the diversity of animal hosts. This highlights the importance of regions with greater diversity, as they often occur a higher incidence of tick-borne virus cases, which has implications for public health. We believe that this mechanism not only explains the relationship between ecoclimate factors and tick virome but also offers valuable insights for future research.

Our findings revealed that climate-related factors appeared to have a stronger association with the evenness of the virome rather than its richness (Fig. 3A, Fig. S3), driving a more evenly distributed virome. This suggests that the viruses carried by ticks may have a more equal opportunity to be involved in spill-over events, potentially favouring their successful establishment in multiple animal hosts. This finding could potentially explain the phenomenon of tick-borne viruses being more inclined to act as generalists in infecting vertebrate hosts, which results in their relatively higher genetic diversity compared to other arthropods, such as mosquitoes [43, 44]. Furthermore, we observed that regions with higher virome diversity in ticks had a greater occurrence of tick-borne pathogen (TBP) infections, underscoring the public health significance of climate-induced changes in the tick virome. Wellfleet Bay virus like showed potential antipathogen capabilities, as it was significantly associated with other resident viruses and highly abundant in regions with lower virome diversity. However, further investigation is needed to explore this potentiality in more depth.

An expanding diversity of tick-borne viruses spread more uniformly across the geographic distribution of their vector, coupled with the predicted temperature-induced changes in host ecology likely increasing host-seeking and an expanded invasive range, together indicated that *H. longicornis* epitomized a significant and burgeoning risk to global public health. As the strong association between climate factors and virome diversity indicated from *H. longicornis* in China, the prediction was conducted to forecast more problem areas in the rest regions worldwide and in the future. Furthermore, it is likely that this risk will be shared unevenly across geographic regions from the prediction results, and developing nations located in emergent hotspots will bear a disproportionate amount of risk. For example, the eastern coastline of China had lower virome diversity, contrast to coastal areas of India, Malaysia, Indonesia, Australia, Mexico, the United States, South America, and Southern Africa predicted to had higher virome diversity. This was consistent with the higher precipitation in coastal versus interior regions of China and the negative association between precipitation and vertebrate-associate virome diversity detected in the GAM analysis (Fig. S16) [45, 46]. Another observation was that tropical and subtropical regions, particularly across South America, Africa, and Asia, were likely to have the highest vertebrate-associated virome diversity (Fig. 5A, Fig. S4-S8, Table S7), and previous research suggested these areas to be more prone to viral emergence [47]. *H. longicornis* has not been reported from tropical localities to date. Nonetheless, previous results predicted that tropical regions are suitable habitats. *H. longicornis*’s invasive characteristics and broad climate envelope indicated that it might become established in these regions in the future [48, 49]. Indeed, it might have been already present in the tropics but unreported, similar to the finding that *H. longicornis* had been an established species in New Jersey, United States for at least several years despite only being publicly reported in 2017 [14, 16]. Although the virome data used for training the predication model were only taken from China, which may not be sufficient to represent the situation in other areas, a limited number of datasets available from the SRA database have been downloaded and applied for verification. They were identified to be low-diversity regions, both from the actual and predicted diversity, which indicated the consistency of identifying diversity levels for these regions from the prediction. Regardless, the finding suggests that tropical regions and some coastal regions are potential problem hotspots of high–tick-borne virome diversity; thus, allocation of surveillance resources are warranted to better understand the possibility of viral spillover events in these areas.

Climate scenarios are developed based on different assumptions about future greenhouse gas emissions, socioeconomic factors, and climate system responses. These scenarios provide a range of possible future climate conditions, considering a wide range of uncertainties. In this study we used SSP2.6, SSP4.5, and SSP8.5, which represent low, medium, and high emissions scenarios. The climate model (CNRM-CM6–1-HR) was used here, which simulated a global mean warming close to historical observations [36]. To make the developed DL model more precise and capture the genuine climate impact on viromes, we utilized actual and specific climatic data from meteorological observation stations to train the DL model. For global predictions, we employed climatic data from CMIP6, which has a resolution of 1 value per 50 km × 50 km. If we use climatic data from CMIP6 to train our DL model could result in most of our collected samples sharing the same climatic data but with different Shannon index values, potentially leading to model distortion. We acknowledge that this approach introduces potential biases that could result in disparities between the predicted and actual values. The future climate data predicted from this climate model were used to explore their impact on virome over the time and suggested that most regions (81.9%) were expected to have increased tick-borne virome diversity under the highest carbon dioxide emission scenario (SSP8.5) in 2030, (Fig. 6, Table S8). The use of climate scenarios helps address the inherent uncertainty associated with predicting future climate conditions accurately and provides a more comprehensive understanding of the potential range of climate outcomes on tick virome.

There are several limitations in this study. First, the viromes dataset used in this study is limited to samples collected from China, which may not be representative enough for training models and making global projections. Second, due to the limited sampling time (only 39 sampling time points), we were unable to conduct time-series predictions. The meteorological factors were averaged using 4-year monthly data to mitigate the temporal effects in the climate model predictions, which could potentially result in underestimations or overestimations. Third, the association between pathogenic viruses and antiviral immunity genes might be biased by human pathogenic viruses. In Fig. 4A, the relatively lower explained variance of 5% suggests that the association may have been underestimated due to the unexplored aspects of antiviral immunity. However, that antiviral immunity showed a higher explained variance of 26.4% for pathogenic abundance in Fig. 4A. Updating the database to include a broader range of immunity genes may lead to a more precise understanding of the association.

Generally, the results in this study are in keeping with the putative benefits that higher temperatures convey to tick-borne virus-vector dynamics and suggest that global warming may induce more frequent spillover events and thus emergence or re-emergence of more tick-borne viruses. Policies that constrain carbon output and limit carbon dioxide emissions to lower trajectories might reduce this threat. Given the significant impact of climate on the tick virome and the resulting potential for cross-transmission events, it is imperative to extend this study to other invasive species (such as *Ixodes scapularis*, *Ixodes ricinus*, and *Rhipicephalus sanguineus*) to assess their viromes under changing ecological conditions. This research will help clarify the level of risk associated with emerging tick-borne virus outbreaks.

## Supplementary Material

Supplementary_Data_wrae087

## Data Availability

The meta-transcriptomic data have been deposited to SRA under Bio-project PRJNA841744, and the assembled virus sequences have been submitted to GenBank (accession no. ON746331-ON746566, ON811696-ON813070, ON811604-ON811608, ON872591-ON872654). The related abundance tables and metadata were provided as Supplementary table 1. All methods and analysis source code were uploaded to GitHub (https://github.com/patience111/climateChange_tickVirusDiversity) and included the MIT license. The Zenodo DOI (https://zenodo.org/doi/10.5281/zenodo.10889637) was created as a new release and please refer to the following link: https://github.com/patience111/climateChange_tickVirusDiversity/releases/tag/v1.0.0
