# Supplementary material for: Ecoclimate drivers shape virome diversity in a globally invasive tick species
Source: ISME J. 2024 May 15;18(1):wrae087. doi: 10.1093/ismejo/wrae087 (PMC11187987; doi:10.1093/ismejo/wrae087)
Supplement: Supplementary_Data_wrae087 [file supplementary_data_wrae087.zip › Supplementary figure and Table legends.docx]

**Supplementary figure legends**

**Figure S1** Number of samples of each ecotype in different groups of the geographic closeness to coastline by 200 km.

**Figure S2** Linear correlation of all factors in the study. The upper panel shows the correlation between the paired variables; the lower presents the scatter plots of the paired variables (x-axis for the top headers and y-axis for the right headers); the diagonal shows the continuous density plots of the variables.

**Figure S3** (A) Linear regression between Shannon index and Simpson E. (B) Linear regression between Shannon index and McIntosh E. (C) Linear regression between Shannon index and Chao1. (D) Linear regression between Shannon index and ACE.

**Figure S4** Global map of predicted vertebrate associated virome diversity under SSP2.6 in 2019.

**Figure S5** Global map of predicted vertebrate associated virome diversity under SSP8.5 in 2019.

**Figure S6** Global map of predicted vertebrate associated virome diversity under SSP2.6 in 2030.

**Figure S7** Global map of predicted vertebrate associated virome diversity under SSP4.5 in 2030.

**Figure S8** Global map of predicted vertebrate associated virome diversity under SSP8.5 in 2030.

**Figure S9** Global map of the difference of predicted vertebrate associated virome diversity between 2019 and 2030 under SSP4.5.

**Figure S10** Global map of predicted vertebrate associated virome diversity under SSP2.6 in 2040.

**Figure S11** Global map of predicted vertebrate associated virome diversity under SSP4.5 in 2040.

**Figure S12** Global map of predicted vertebrate associated virome diversity under SSP8.5 in 2040.

**Figure S13** Global map of predicted vertebrate associated virome diversity under SSP2.6 in 2050.

**Figure S14** Global map of predicted vertebrate associated virome diversity under SSP4.5 in 2050.

**Figure S15** Global map of predicted vertebrate associated virome diversity under SSP8.5 in 2050.

**Figure S16** Association of virome diversity, mean temperature, and mean precipitation by a 3D plot. Dots, representing different sample libraries, are colored by the different coastline groups.

**Supplementary tables**

**Table S1** Meta-information, virome diversity, and pathogenic viral abundance for each library included in this study.

**Table S2** Significance (adjusted *P* value by BH method) of statistical comparison of vertebrate associated virome diversity among groups in the six divisions of distance from the coastline, and the explained variation of each grouping method in the GAM.

**Table S3** Significance (adjusted *P* value by BH method) of pairwise comparisons of vertebrate associated virome diversity between groups clustered by every 100 km distance away from the coastline using Wilcoxon signed-rank test. Significant *P* values (at 95% confidence) are highlighted in red.

**Table S4** General linear regression analysis for different virome evenness indices and richness indices against Shannon index.

**Table S5** Pearson coefficients of the relative abundance of viruses against Shannon index of vertebrate associated virome and the relative abundance of Wellfleet Bay virus like.

**Table S6** Explained deviance of GAM models for viral abundance and eco-climate factors.

**Table S7** Vertebrate associated virome diversity in Korea and USA using public RNA-sequencing data.

**Table S8** Vertebrate associated virome diversity for predicted high risk regions in 2019 under SSP4.5.

**Table S9** Difference of vertebrate associated virome diversity from 2030 to 2019 under three SSP scenarios.
