# Supplementary material for: Ecoclimate drivers shape virome diversity in a globally invasive tick species
Source: ISME J. 2024 May 15;18(1):wrae087. doi: 10.1093/ismejo/wrae087 (PMC11187987; doi:10.1093/ismejo/wrae087)
Supplement: Supplementary_Data_wrae087 [file supplementary_data_wrae087.zip › Supplementary_table_2-9.docx]

**Table S2** Significance (adjusted *p* value by BH method) of statistical comparison of **v**ertebrate associated virome diversity among groups clustered by six distance cut-offs away from coastline, and the explained deviance of each grouping method by GAM.

| The distance cutoff (distance to coastline) for grouping | *p* value  (Kruskal-Wallis test) | GAM deviance explained |
| --- | --- | --- |
| 100 km | 2.31E-16 | 42.9% |
| 200 km | 2.20E-16 | 39.6% |
| 300 km | 8.48E-15 | 30.5% |
| 400 km | 2.20E-16 | 37.9% |
| 500 km | 2.24E-10 | 20.7% |
| 600 km | 5.41E-10 | 18.1% |

**Table S3** Significance (adjusted *p* value by BH method) of pairwise comparison on the **v**ertebrate associated virome diversity between groups clustered by every 100km distance away from the coastline using Wilcoxon signed-rank test. Significant *p* values (at 95% confidence) are highlighted in red.

|  | 0<$x$≤100km | 100<$x$≤200km | 200<$x$≤300km | 300<$x$≤400km | 400<$x$≤500km | 500<$x$≤600km | 600<$x$≤700km | 700<$x$≤800km | 800<$x$≤900km | 900<$x$≤1,000km | 1,000<$x$≤1,100km |
| --- | --- | --- | --- | --- | --- | --- | --- | --- | --- | --- | --- |
| 100<$x$≤200km | 0.89 | - | - | - | - | - | - | - | - | - | - |
| 200<$x$≤300km | 0.60 | 0.61 | - | - | - | - | - | - | - | - | - |
| 300<$x$≤400km | 0.33 | 0.20 | 0.72 | - | - | - | - | - | - | - | - |
| 400<$x$≤500km | 0.00 | 0.00 | 0.23 | 0.20 | - | - | - | - | - | - | - |
| 500<$x$≤600km | 0.01 | 0.03 | 0.20 | 0.46 | 0.89 | - | - | - | - | - | - |
| 600<$x$≤700km | 0.00 | 0.00 | 0.02 | 0.14 | 0.53 | 0.75 | - | - | - | - | - |
| 700<$x$≤800km | 0.10 | 0.13 | 0.39 | 0.52 | 0.51 | 0.61 | 0.20 | - | - | - | - |
| 800<$x$≤900km | 0.20 | 0.32 | 0.96 | 0.20 | 0.01 | 0.12 | 0.00 | 0.20 | - | - | - |
| 900<$x$≤1,000km | 0.00 | 0.00 | 0.25 | 0.20 | 0.20 | 0.46 | 0.00 | 0.52 | 0.11 | - | - |
| 1,000<$x$≤1,100km | 0.00 | 0.01 | 0.23 | 0.25 | 0.20 | 0.46 | 0.00 | 0.61 | 0.18 | 0.53 | - |
| 1,100<$x$≤1,200km | 0.00 | 0.01 | 0.19 | 0.41 | 0.61 | 0.61 | 0.80 | 0.39 | 0.05 | 0.22 | 0.20 |

**Table S4** General linear regression analysis for different virome evenness index and richness index against Shannon index.

| GLM modelling | Dependent variable | Independent variable | Coefficients | *p* value | *R*^2^ |
| --- | --- | --- | --- | --- | --- |
| Model 1 | Shannon index | Simpson_E | 14.02 | <2E-16 | 0.82 |
|  |  | Chao1 | 0.04 | <2E-16 |  |
| Model 2 | Shannon index | Simpson_E | 13.46 | <2E-16 | 0.71 |
|  |  | ACE | 0.01 | <2E-16 |  |
| Model 3 | Shannon index | McIntosh_E | 3.49 | <2E-16 | 0.65 |
|  |  | Chao1 | 0.02 | 5.42E-9 |  |
| Model 4 | Shannon index | McIntosh_E | 3.52 | <2E-16 | 0.62 |
|  |  | ACE | 5.28E-3 | 9.18E-4 |  |

**Table S5** Pearson coefficients of the relative abundance of viruses against Shannon index of vertebrate associated virome and the relative abundance of Wellfleet bay virus.

| Objective1 | Objective2 | Correlation | *p* value |
| --- | --- | --- | --- |
| Shannon index | Wellfleet bay virus | -0.75 | 4.25E-44 |
| Shannon index | Tacheng tick virus 7 | -0.16 | 1.53E-02 |
| Shannon index | SFTS phlebovirus | 0.13 | 4.61E-02 |
| Shannon index | Dabieshan tick virus | 0.13 | 4.43E-02 |
| Shannon index | Huangpi tick virus 3 | 0.15 | 2.52E-02 |
| Shannon index | Tjuloc virus | 0.16 | 1.62E-02 |
| Shannon index | Radi vesiculovirus | 0.18 | 6.67E-03 |
| Shannon index | Wuhan louse fly virus 3 | 0.22 | 6.37E-04 |
| Shannon index | Wuhan ant virus | 0.44 | 1.07E-12 |
| Shannon index | South bay virus | 0.53 | 8.98E-19 |
| Shannon index | Diachasmimorpha longicaudata rhabdovirus | 0.55 | 1.37E-20 |
| Shannon index | Araguari virus | 0.56 | 6.73E-21 |
| Shannon index | Bole tick virus 1 | 0.57 | 3.92E-22 |
| Shannon index | Rhipicephalus associated rhabdo-like virus | 0.59 | 6.83E-24 |
| Shannon index | Drosophila busckii rhabdovirus | 0.64 | 8.50E-29 |
| Shannon index | Nayun tick rhabdovirus | 0.69 | 2.22E-35 |
| Shannon index | Quaranfil quaranjavirus | 0.70 | 9.11E-37 |
| Shannon index | Tacheng tick virus 3 | 0.72 | 1.18E-38 |
| Wellfleet bay virus | Nayun tick rhabdovirus | -0.48 | 4.17E-15 |
| Wellfleet bay virus | Tacheng tick virus 3 | -0.48 | 5.60E-15 |
| Wellfleet bay virus | Dabieshan tick virus | -0.47 | 1.97E-14 |
| Wellfleet bay virus | Quaranfil quaranjavirus | -0.43 | 3.61E-12 |
| Wellfleet bay virus | Drosophila busckii rhabdovirus | -0.39 | 5.40E-10 |
| Wellfleet bay virus | South bay virus | -0.36 | 1.41E-08 |
| Wellfleet bay virus | Diachasmimorpha longicaudata rhabdovirus | -0.36 | 1.76E-08 |
| Wellfleet bay virus | Bole tick virus 1 | -0.35 | 3.60E-08 |
| Wellfleet bay virus | Rhipicephalus associated rhabdo-like virus | -0.34 | 6.80E-08 |
| Wellfleet bay virus | Araguari virus | -0.33 | 2.23E-07 |
| Wellfleet bay virus | Wuhan ant virus | -0.26 | 5.83E-05 |
| Wellfleet bay virus | Bole tick virus 2 | -0.16 | 1.40E-02 |
| Wellfleet bay virus | Wuhan louse fly virus 3 | -0.16 | 1.42E-02 |

**Table S6** Explained deviance of GAM models for viral abundance and eco-climate factors.

| Virus | Sample Number | Explained Deviance (%) | | | | | | | | |
| --- | --- | --- | --- | --- | --- | --- | --- | --- | --- | --- |
|  |  | All factors | Climate factors | Ecotype | Coastline Group | Gender | Feeding animal | Livestock animal | All mammals | Anti_viral immunity |
| Wellfleet Bay virus | 237 | 95.20 | 89.20 | 2.18 | 27.40 | 6.91 | 0.18 | 22.40 | 19.30 | 9.78 |
| Tacheng Tick Virus 3 | 238 | 55.80 | 45.70 | 0.01 | 29.70 | 0.00 | 0.04 | 5.89 | 7.41 | 3.31 |
| Nayun tick rhabdovirus | 234 | 70 | 50.40 | 1.28 | 23.10 | 1.19 | 2.19 | 6.18 | 11 | 7.18 |

**Table S7** Vertebrate associated virome diversity in Korea and USA using public RNA-sequencing data.

| SRA accession number | Collection date | Geographic location | Latitude and Longitude | Actual Shannon index | Mean of actual Shannon index | Predicted Shannon index | Mean of predicted Shannon index using all data* |
| --- | --- | --- | --- | --- | --- | --- | --- |
| SRR13962109 | 2019 | South Korea: Sungnam | 37.44N 127.14E | 2.131616 | 2.138199 | 2.923101 | 2.952418 (2019) |
| SRR13962108 | 2019 | South Korea: Sungnam | 37.44N 127.14E | 2.144782 |  |  |  |
| SRR12375045 | 2019/7/30 | South Korea:Chuncheon | 38.02N 127.38E | 1.942215 | 2.421212 | 2.719797 | 2.952418 (2019) |
| SRR12375044 | 2019/7/30 | South Korea:Chuncheon | 38.02N 127.38E | 2.227161 |  |  |  |
| SRR12375043 | 2019/7/30 | South Korea:Chuncheon | 38.02N 127.38E | 3.018257 |  |  |  |
| SRR12375042 | 2019/7/30 | South Korea:Chuncheon | 38.02N 127.38E | 2.497217 |  |  |  |
| SRR15089914 | 2021/6/9 | USA: New Jersey, New Brunswick | 40.49N 74.45W | 1.795985 | 1.754844 | 2.730282 | 2.953855 (2021) |
| SRR15089913 | 2021/6/9 | USA: New Jersey, New Brunswick | 40.49N 74.45W | 1.713702 |  |  |  |

*Mean of predicted Shannon index using all data from deep learning prediction of the corresponding sampling year, as shown in Brackets. Both the actual Shannon index and the predicted one were lower than the corresponding overall mean Shannon index (averaged Shannon index of all locations).

**Table S8** Vertebrate associated virome diversity for predicted risky regions in 2019 under SSP 4.5.

| Region | Mean | Median | Minimum | Maximum | 95% Confidential Interval |
| --- | --- | --- | --- | --- | --- |

| India | 3.1668 | 3.1814 | 2.6735 | 3.2851 | [3.0267, 3.3069] |
| --- | --- | --- | --- | --- | --- |
| Golden triangle area | 3.1613 | 3.1647 | 2.9999 | 3.2577 | [3.0659, 3.2565] |
| Southeast Asia | 3.0367 | 3.0381 | 2.6000 | 3.3520 | [2.8376, 3.2357] |
| Western coastline of USA | 3.0457 | 3.0412 | 2.9832 | 3.1055 | [2.9979, 3.0934] |
| Northern coastline of Australia | 3.1286 | 3.1161 | 2.9308 | 3.3060 | [2.9705, 3.2867] |
| Coast area of Mexico | 3.1286 | 3.1161 | 2.9308 | 3.3060 | [2.9705, 3.2867] |
| South America | 3.1653 | 3.1708 | 2.9369 | 3.2597 | [3.0732, 3.2574] |
| Southern Africa | 3.1798 | 3.1847 | 2.9632 | 3.2609 | [3.0944, 3.2651] |
| Eastern Russia | 2.7555 | 2.7541 | 2.6253 | 2.8753 | [2.6552, 2.8558] |
| Qinghai-Tibet | 2.8480 | 2.7974 | 2.6319 | 3.2062 | [2.5647, 3.1313] |
| **Global** | **2.9524** | **2.9417** | **2.5005** | **3.3565** | **[2.9508, 2.9540]** |

Note: High-risk regions are highlighted in red, while low-risk region in green.

**Table S9** Difference of vertebrate associated virome diversity from 2030 to 2019 under three SSP scenarios.

|  | SSP | Mean percentage of increased Shannon index | Proportion of locations with increased Shannon index |
| --- | --- | --- | --- |
| Evidence points | SSP2.6 | -0.09% (95%CI: -0.95%, 0.75%) | 41.24% (95%CI: 33.97%, 48.52%) |
|  | SSP4.5 | 0.19% (95%CI: -0.42%, 0.81%) | 71.18% (95%CI: 64.49%, 77.88%) |
|  | SSP8.5 | 0.33% (95%CI: -0.37%, 01.03%) | 81.92% (95%CI: 76.24%, 87.61%) |
| Sampling locations | SSP2.6 | 0.43% (95%CI: 0.044%, 0.99%) | 30.43% (95%CI: 24.59%, 36.28%) |
|  | SSP4.5 | 0.35% (95%CI:0.057%, 0.74%) | 73.91% (95%CI: 68.33%, 79.49%) |
|  | SSP8.5 | 0.33% (95%CI: 0.024%, 0.80%) | 76.09% (95%CI: 70.66%, 81.51%) |
