# Supplementary figures and images for: Ecoclimate drivers shape virome diversity in a globally invasive tick species
Source: ISME J. 2024 May 15;18(1):wrae087. doi: 10.1093/ismejo/wrae087 (PMC11187987; doi:10.1093/ismejo/wrae087)

Virome diversity prediction under SSP2.6: 2040

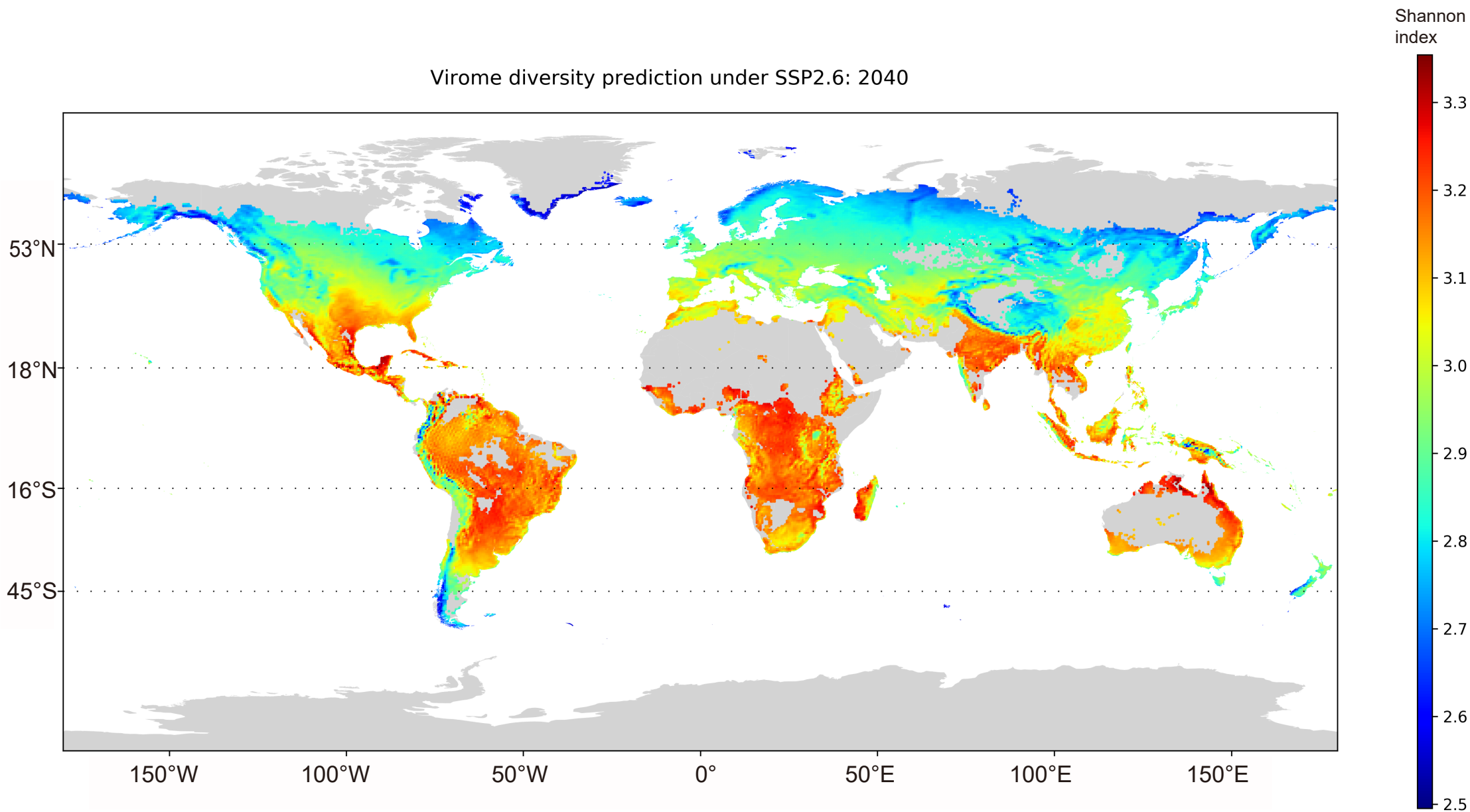

Supplement: Supplementary_Data_wrae087 [file supplementary_data_wrae087.zip › supplementary_figure10.pdf]

Virome diversity prediction under SSP4.5: 2040

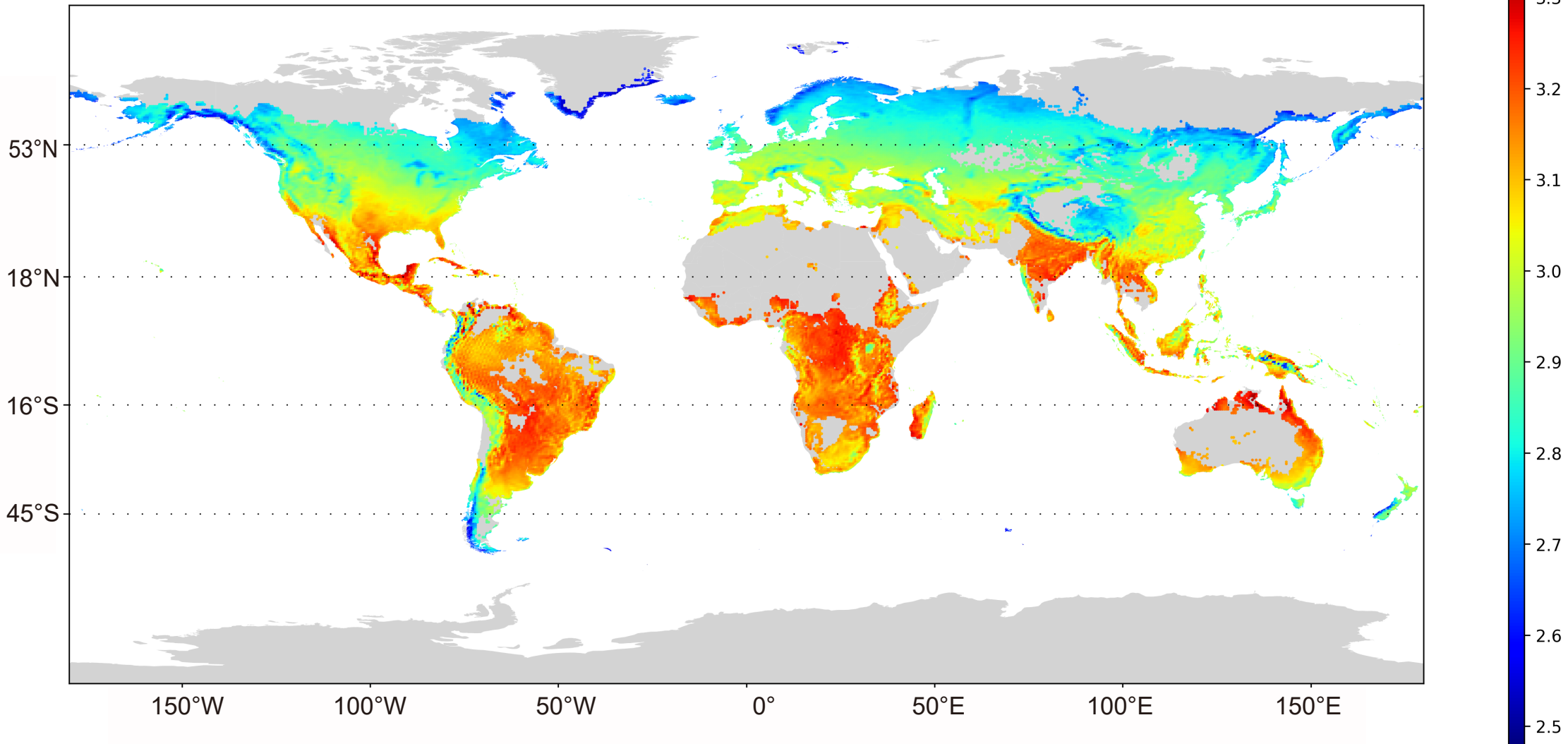

Supplement: Supplementary_Data_wrae087 [file supplementary_data_wrae087.zip › supplementary_figure11.pdf]

Virome diversity prediction under SSP8.5: 2040

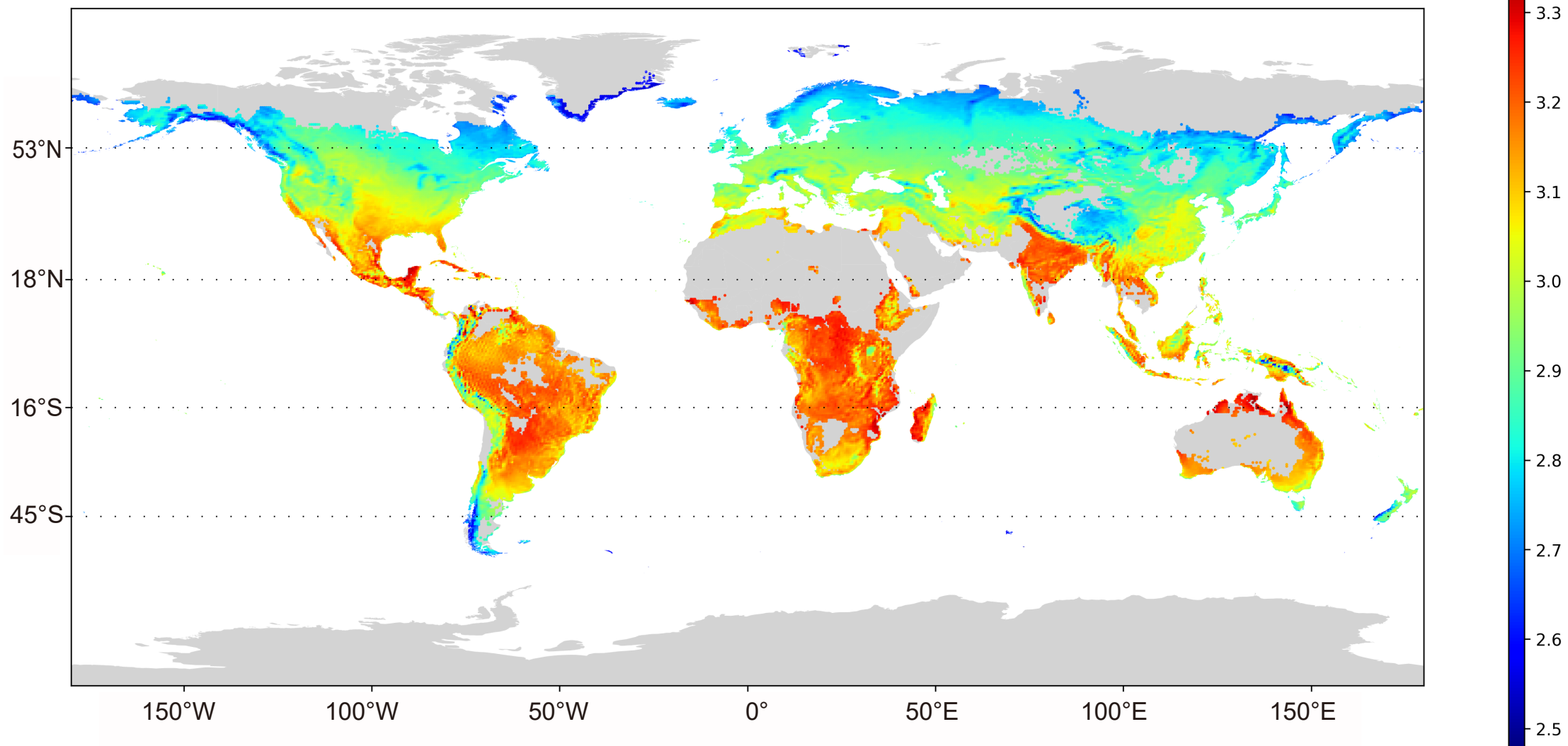

Supplement: Supplementary_Data_wrae087 [file supplementary_data_wrae087.zip › supplementary_figure12.pdf]

Virome diversity prediction under SSP2.6: 2050

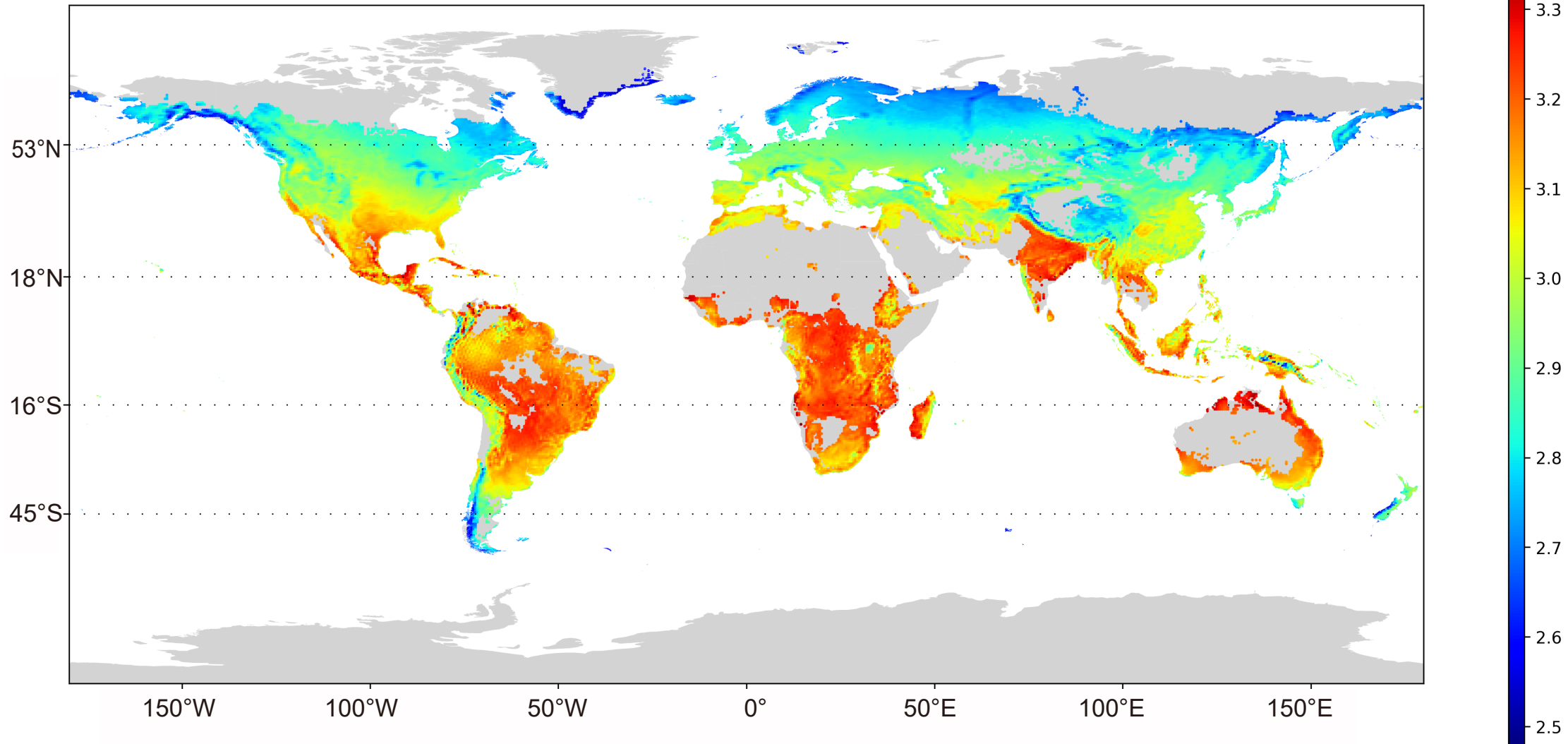

Supplement: Supplementary_Data_wrae087 [file supplementary_data_wrae087.zip › supplementary_figure13.pdf]

Virome diversity prediction under SSP4.5: 2050

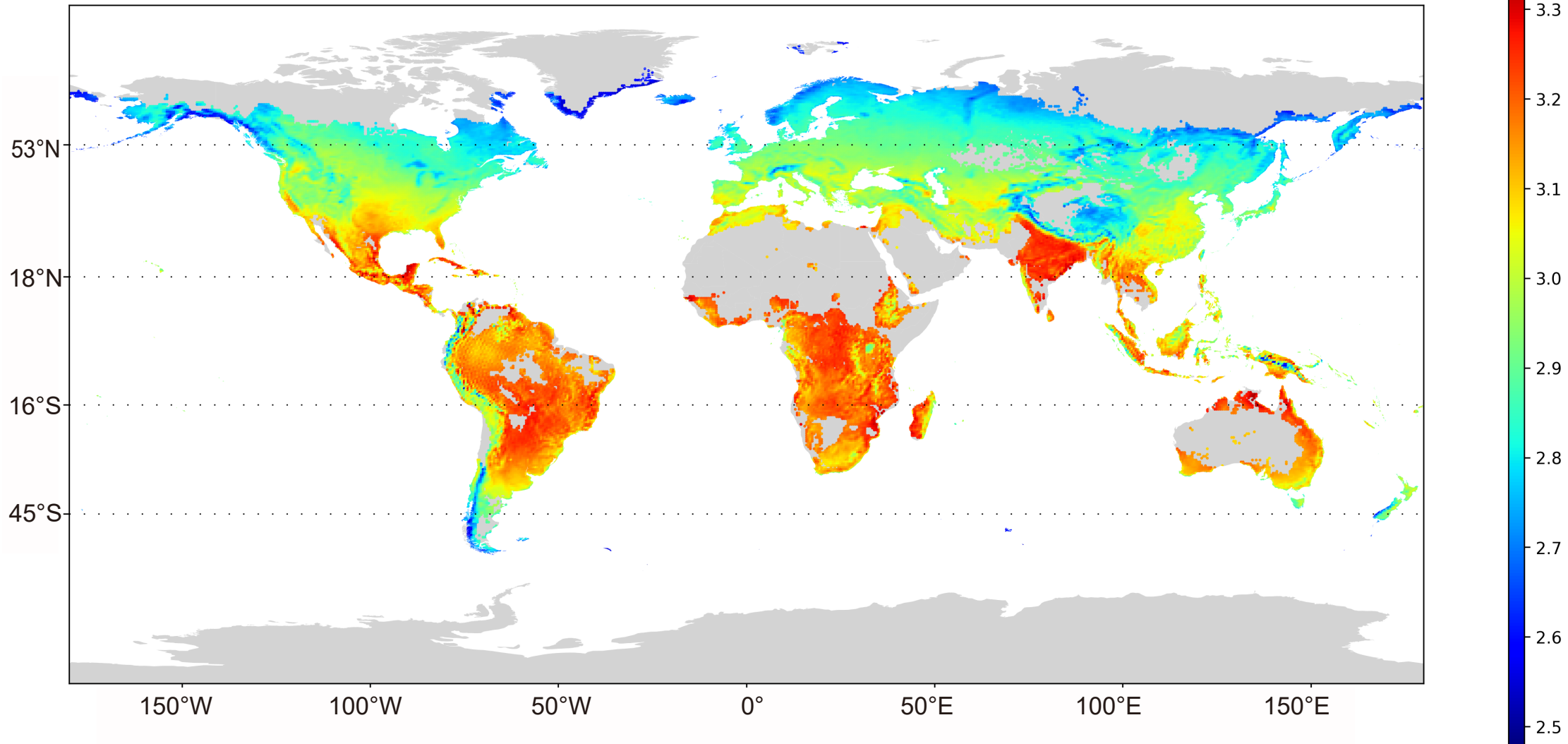

Supplement: Supplementary_Data_wrae087 [file supplementary_data_wrae087.zip › supplementary_figure14.pdf]

Virome diversity prediction under SSP8.5: 2050

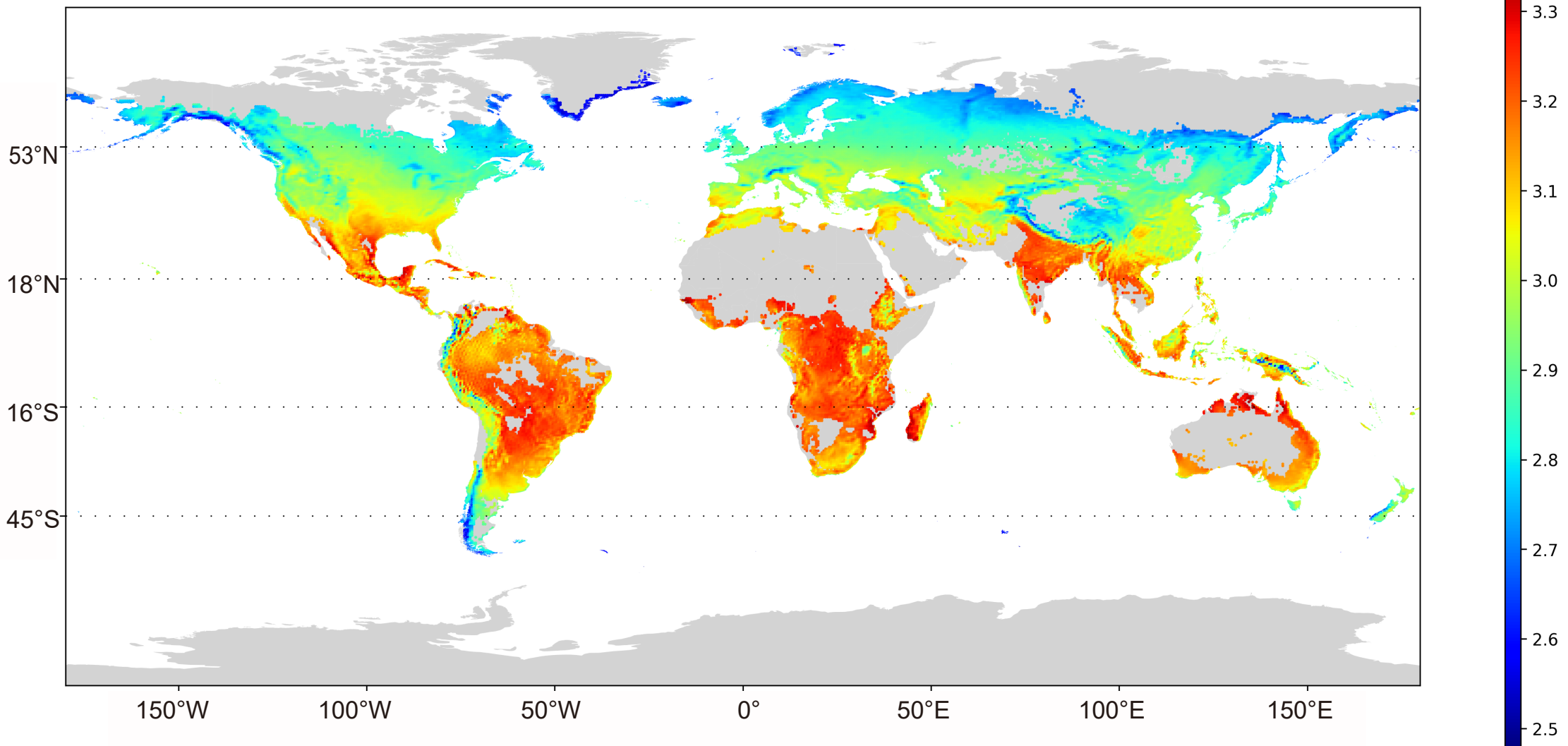

Supplement: Supplementary_Data_wrae087 [file supplementary_data_wrae087.zip › supplementary_figure15.pdf]

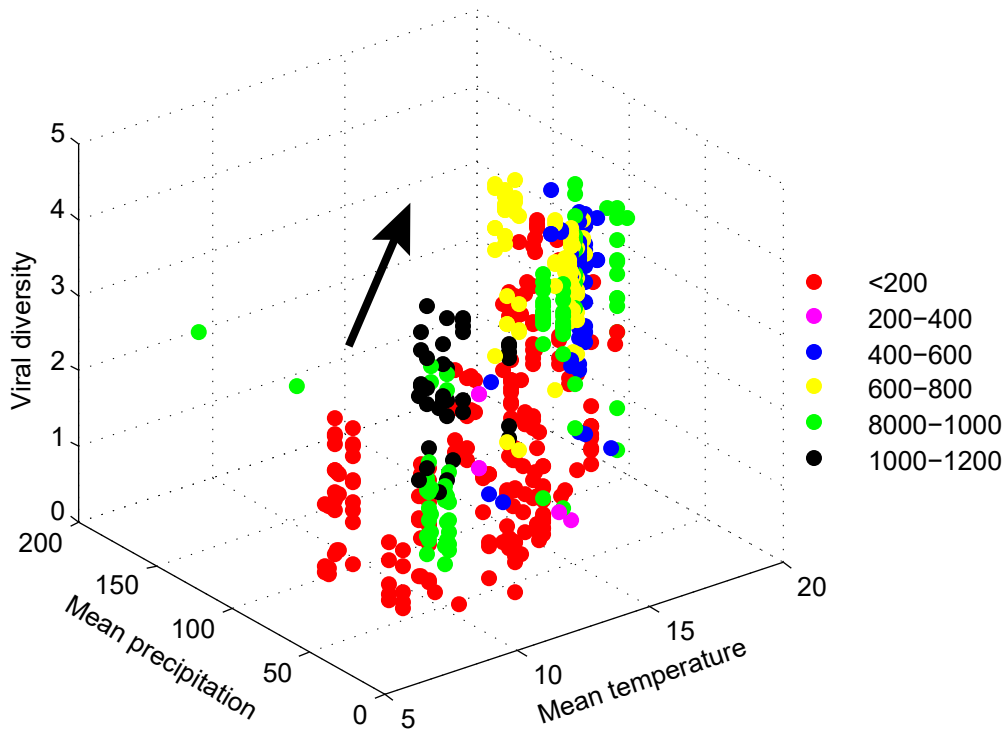

Supplement: Supplementary_Data_wrae087 [file supplementary_data_wrae087.zip › supplementary_figure16.pdf]

Number of Ecology Type

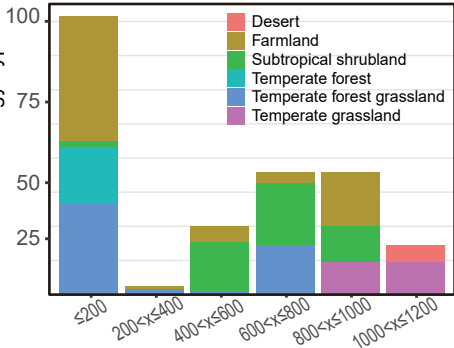

Supplement: Supplementary_Data_wrae087 [file supplementary_data_wrae087.zip › supplementary_figure1_20220814.pdf]

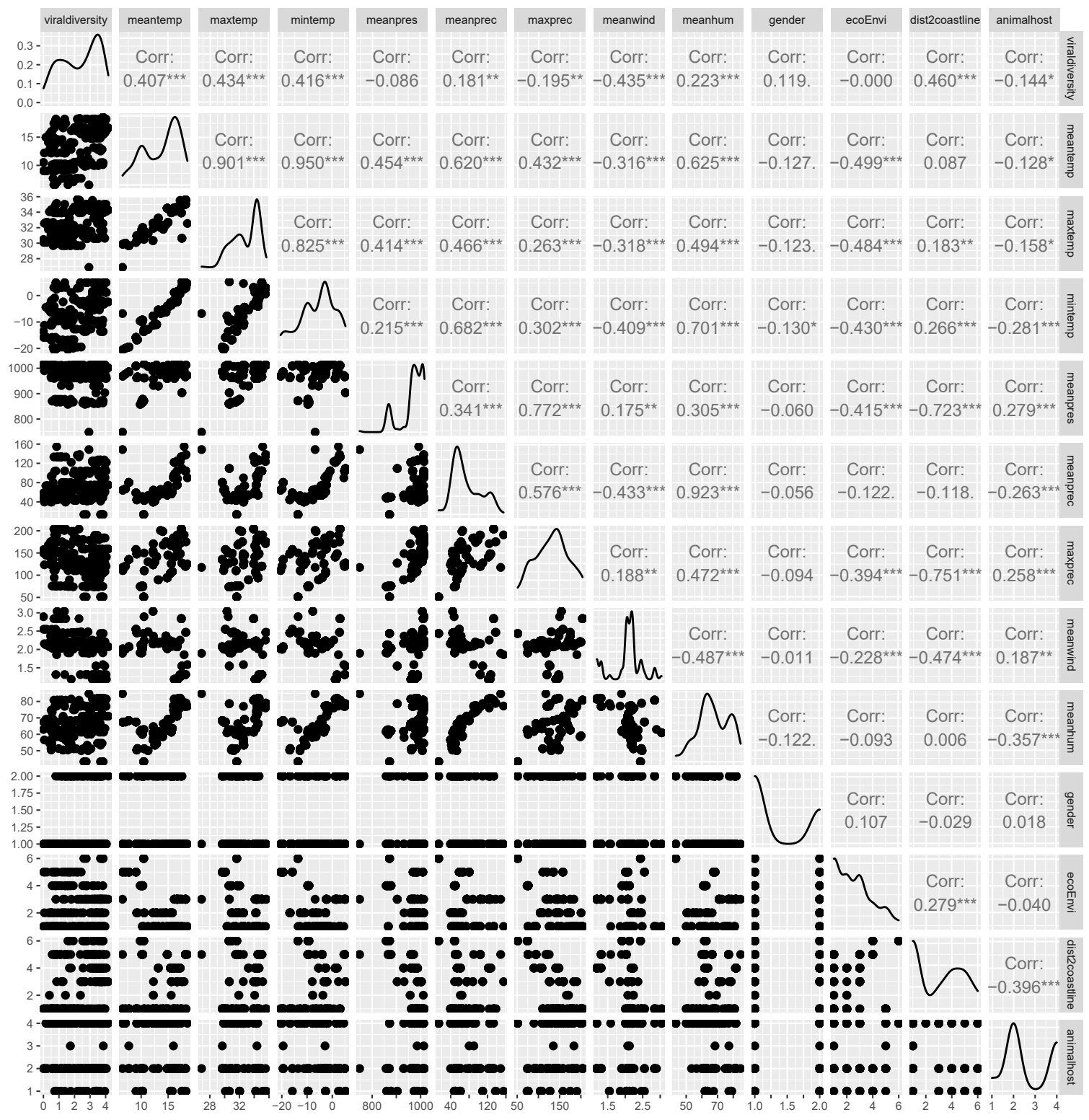

Supplement: Supplementary_Data_wrae087 [file supplementary_data_wrae087.zip › supplementary_figure2_20220717.pdf]

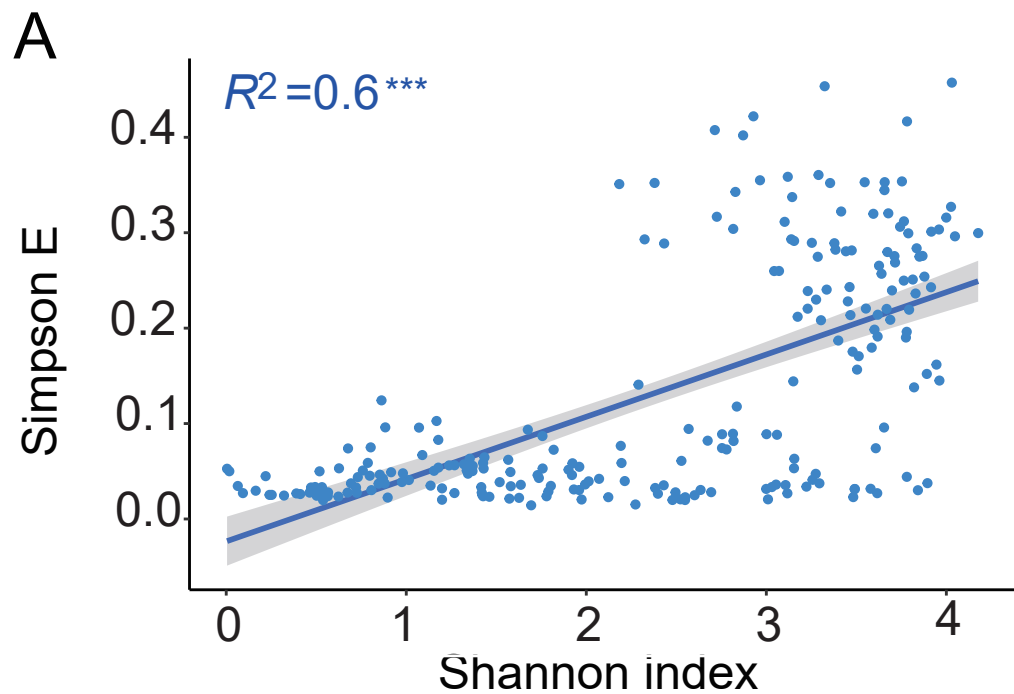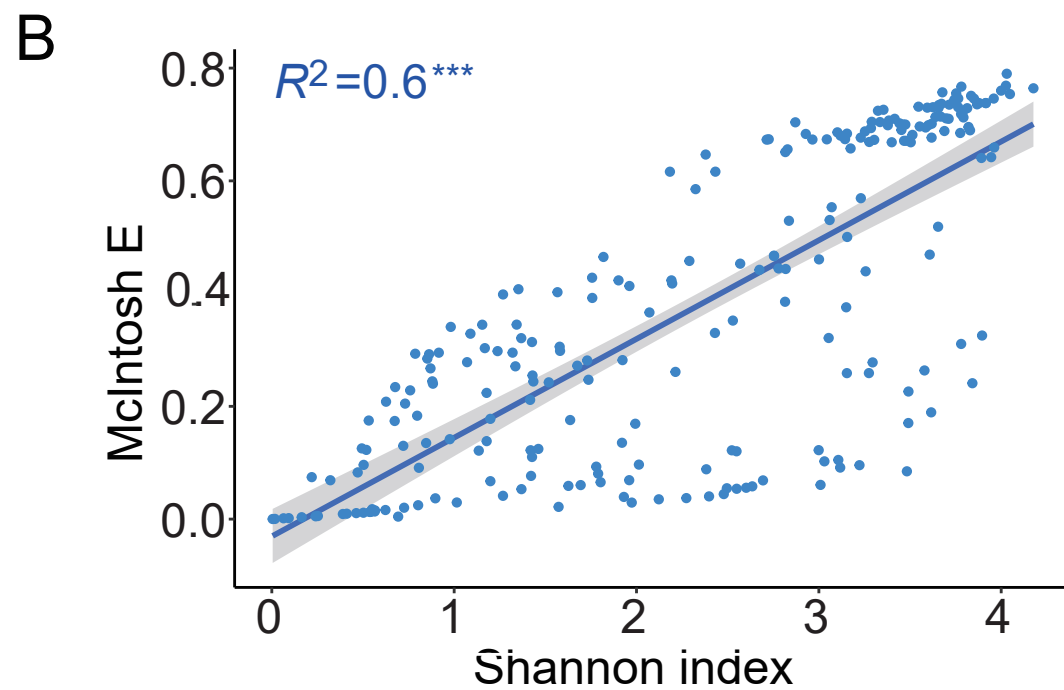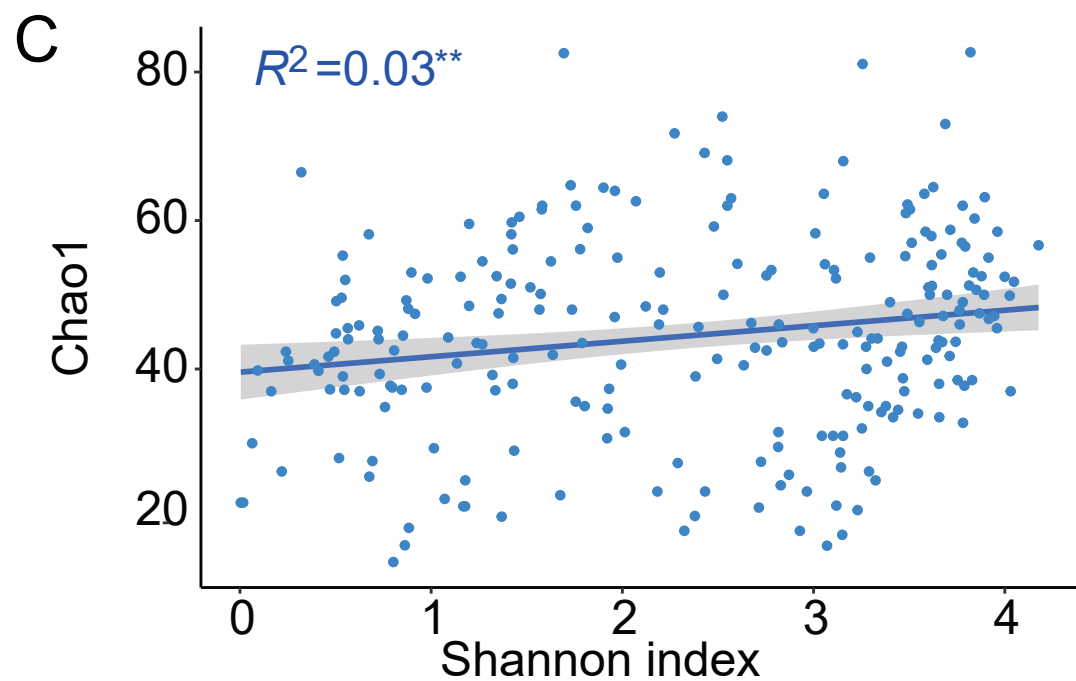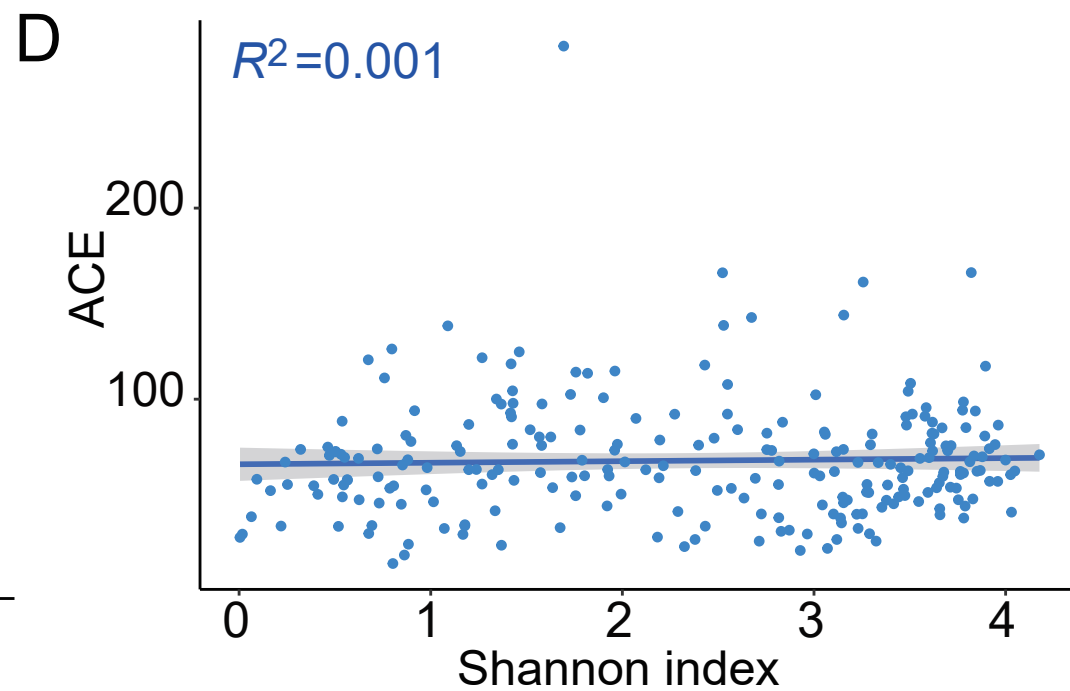

Supplement: Supplementary_Data_wrae087 [file supplementary_data_wrae087.zip › supplementary_figure3_20240328.pdf]

# Virome diversity prediction under SSP2.6: 2019

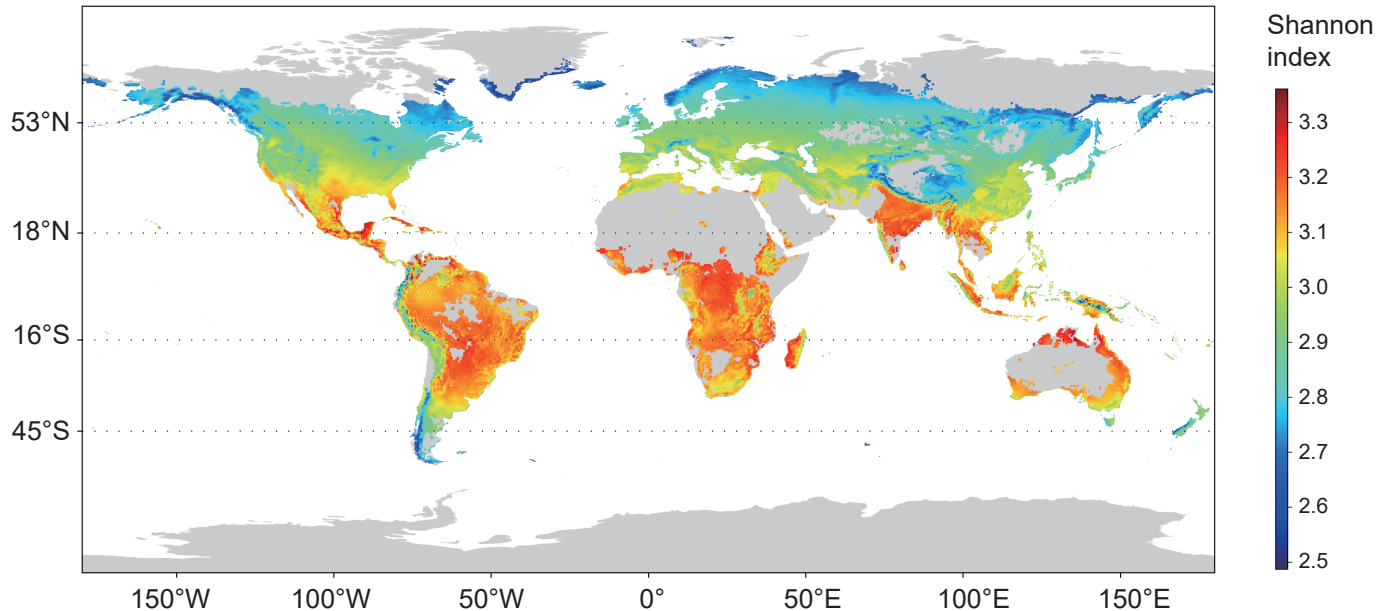

Supplement: Supplementary_Data_wrae087 [file supplementary_data_wrae087.zip › supplementary_figure4_20220915.pdf]

# Virome diversity prediction under SSP8.5: 2019

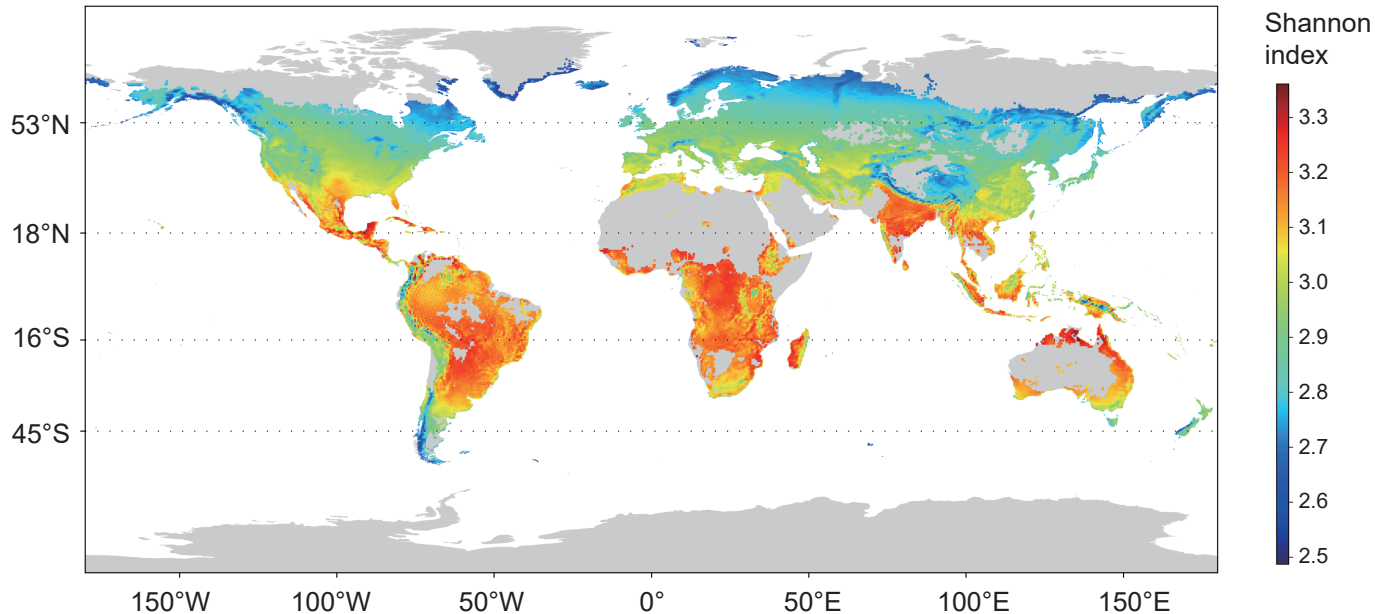

Supplement: Supplementary_Data_wrae087 [file supplementary_data_wrae087.zip › supplementary_figure5_20220915.pdf]

Virome diversity prediction under SSP2.6: 2030

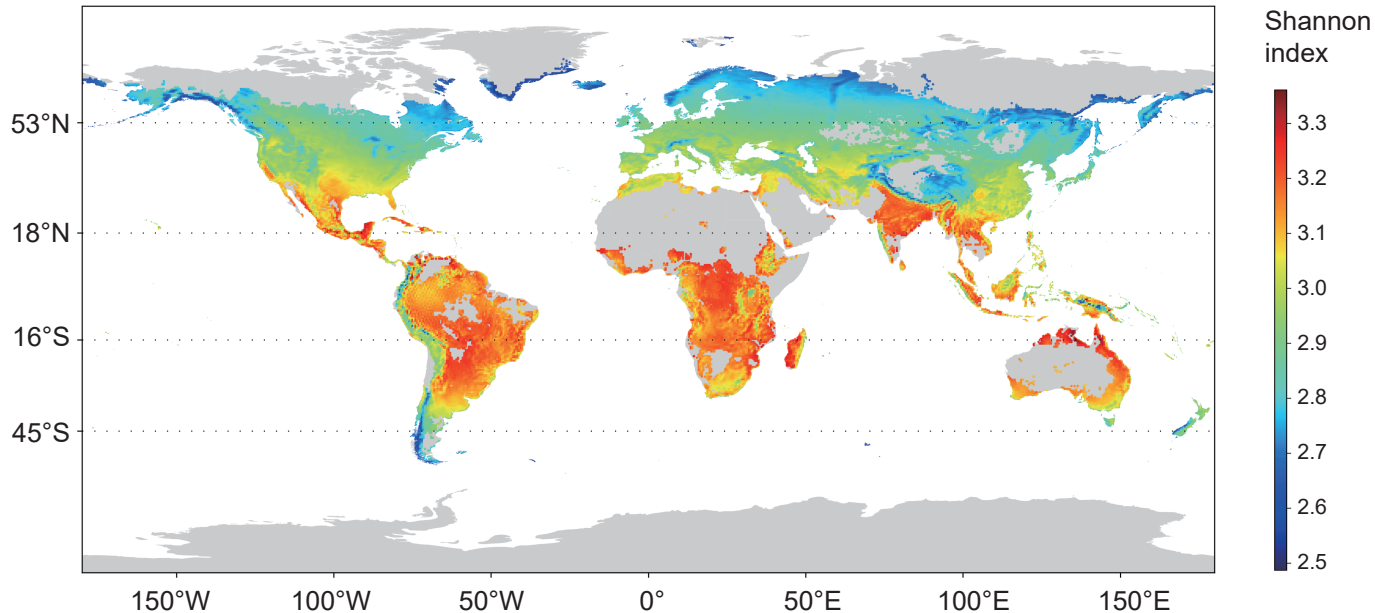

Supplement: Supplementary_Data_wrae087 [file supplementary_data_wrae087.zip › supplementary_figure6_20220915.pdf]

Virome diversity prediction under SSP4.5: 2030

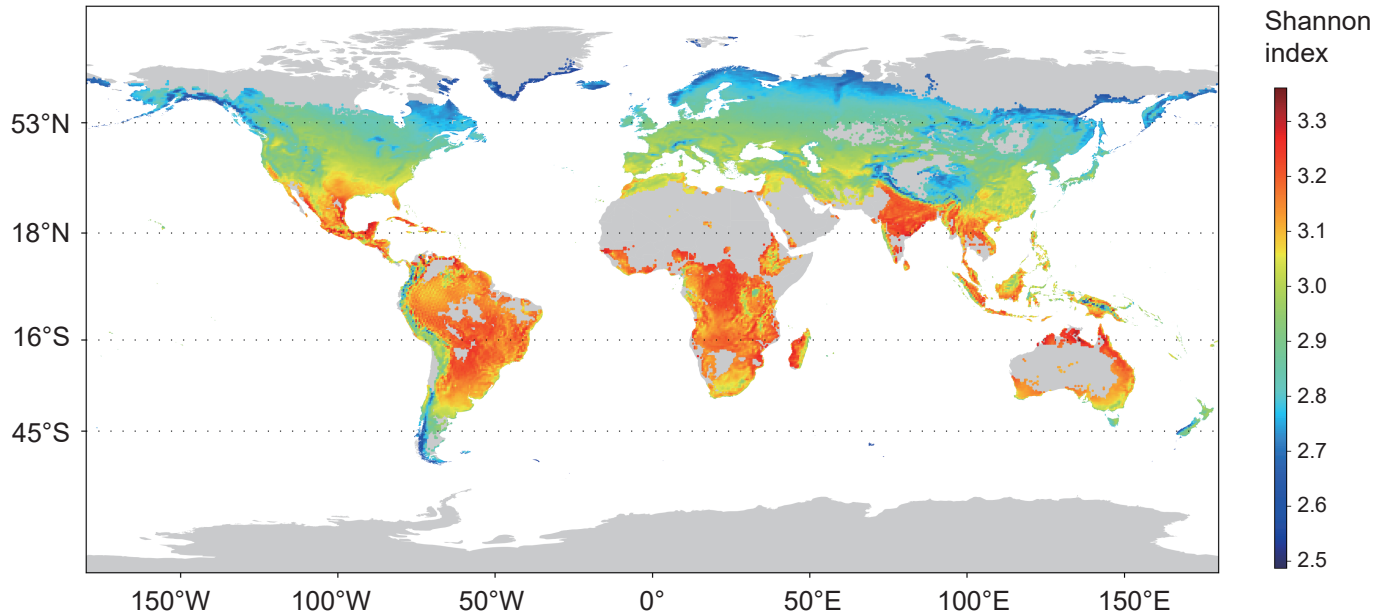

Supplement: Supplementary_Data_wrae087 [file supplementary_data_wrae087.zip › supplementary_figure7_20220915.pdf]

Virome diversity prediction under SSP8.5: 2030

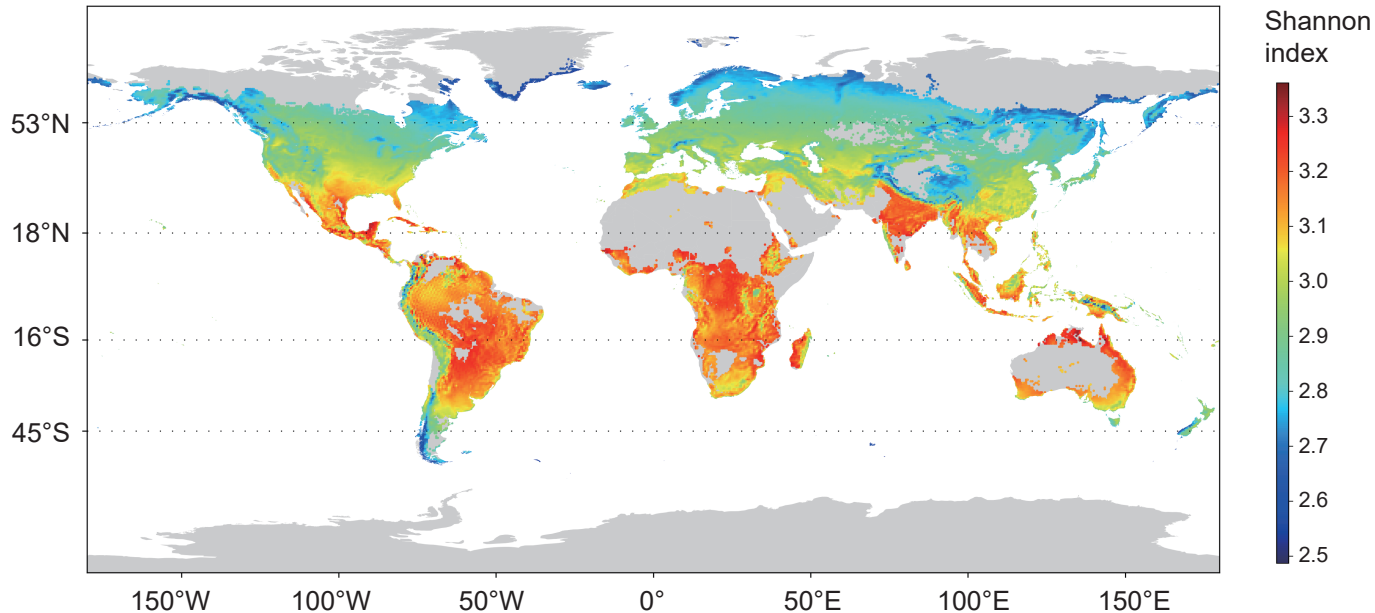

Supplement: Supplementary_Data_wrae087 [file supplementary_data_wrae087.zip › supplementary_figure8_20220915.pdf]

The difference of virome diversity between 2030 and 2019 under SSP4.5

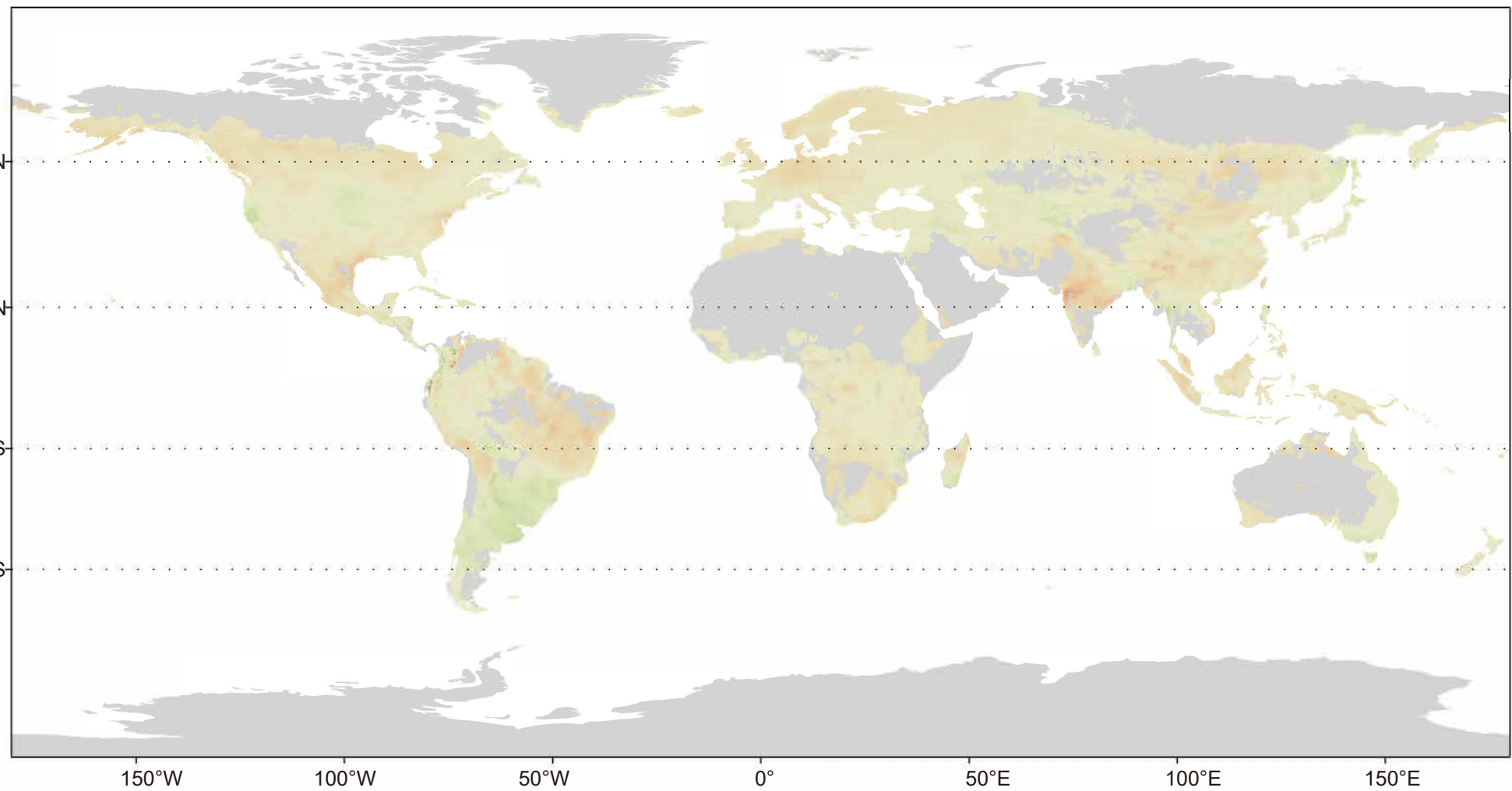

Shannon  
index

0.10

0.05

0.00

-0.05

-0.10

Supplement: Supplementary_Data_wrae087 [file supplementary_data_wrae087.zip › supplementary_figure9.pdf]
